# Surviving Fibroblasts After Treatment With Mitomycin C Exhibit SASP‐Like Phenotype After Glaucoma Filtration Surgery

**DOI:** 10.1111/acel.70680

**Published:** 2026-08-20

**Authors:** Akitoshi Kimura, Tomokazu Fujimoto, Satoshi Iraha, Kei‐Ichiro Yasunaga, Miyuki Inoue‐Mochita, Naofumi Funagura, Shunsuke Tanigawa, Daisuke Kurotaki, Tomoaki Koga, Fumika Watanabe‐Kitamura, Utako Tsutsumi‐Kuroda, Mitsuyoshi Nakao, Toshihiro Inoue

**Affiliations:** ^1^ Department of Ophthalmology, Faculty of Life Sciences Kumamoto University Kumamoto Japan; ^2^ Liaison Laboratory Research Promotion Center, Institute of Molecular Embryology and Genetics Kumamoto University Kumamoto Japan; ^3^ Department of Kidney Development, Institute of Molecular Embryology and Genetics Kumamoto University Kumamoto Japan; ^4^ Laboratory of Chromatin Organization in Immune Cell Development, International Research Center for Medical Sciences Kumamoto University Kumamoto Japan; ^5^ Center for Metabolic Regulation of Healthy Aging, Graduate School of Medical Sciences Kumamoto University Kumamoto Japan; ^6^ Department of Medical Cell Biology, Institute of Molecular Embryology and Genetics Kumamoto University Kumamoto Japan; ^7^ Inomata Eye Clinic Kumamoto Japan; ^8^ Inada Eye Clinic Kumamoto Japan

## Abstract

The clinical outcomes of trabeculectomy, a standard glaucoma filtration surgery (GFS), have dramatically improved with the introduction of mitomycin C (MMC), an antimetabolite widely used to inhibit postoperative scarring. MMC exerts anti‐scarring effects primarily by promoting apoptosis and suppressing fibroblast proliferation at the surgical site. However, despite these beneficial effects, MMC also induces the production of inflammatory cytokines, and its full mechanism of action remains incompletely understood. Moreover, a subset of patients continues to experience poor surgical outcomes even after MMC application, highlighting the need for complementary therapeutic strategies. Comprehensive transcriptomic analysis of postoperative GFS tissues revealed that MMC promoted the expression of genes associated with cellular senescence and inflammatory responses. Supporting this, in vitro experiments suggested that the transcriptional changes were associated with MMC‐induced senescence‐associated secretory phenotype (SASP)‐like changes in the fibroblasts. Single‐cell RNA sequencing (scRNA‐Seq) analyses of postoperative GFS tissues also revealed that fibroblasts after GFS with MMC secrete SASP factors. Furthermore, Cell–Cell communication analysis identified CCL7 as one of the key molecules involved in inflammatory cell migration and the promotion of fibrosis. Importantly, we demonstrated that fibroblasts exhibiting SASP‐like features can be selectively eliminated by a specific senolytic agent, suggesting a novel and promising therapeutic approach that may improve long‐term surgical outcomes after GFS.

## Graphical abstract

Transcriptomic analysis using a rabbit glaucoma filtration surgery model, combined with in vitro experiments on human conjunctival fibroblasts, demonstrates that mitomycin C induces a senescence‐associated secretory phenotype (SASP)‐like state. These SASP‐like fibroblasts were selectively eliminated by the specific senolytic agent, highlighting their potential as a novel therapeutic target. The graphical abstract image was created in BioRender. https://BioRender.com/jo8be38.

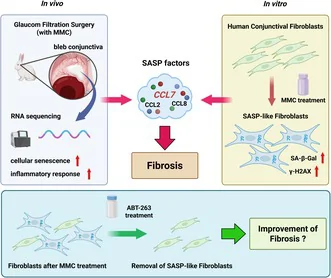

## 1 Introduction

Glaucoma is the leading cause of irreversible blindness worldwide. The global number of people with glaucoma was estimated to be 76.0 million in 2020 and will increase to 111.8 million by 2040 (GBD 2019 Blindness and Vision Impairment Collaborators and Vision Loss Expert Group of the Global Burden of Disease Study 2021; Tham et al. 2014). Thus, improved treatments are urgently required for this condition. The outcomes of trabeculectomy, a standard form of glaucoma filtration surgery (GFS), have dramatically improved with the clinical application of mitomycin C (MMC) and 5‐fluorouracil (Chen 1983; Kitazawa et al. 1991; Kitazawa et al. 1987), the applications of which have been expanded to certain GFS procedures that use devices such as the Ex‐PRESS Glaucoma Filtration Device, Xen Gel Stent, and Preserflo MicroShunt. MMC is an antitumor antibiotic that induces interstrand DNA crosslinks and promotes apoptosis. MMC suppresses cell proliferation, contraction, myofibroblast differentiation, extracellular matrix (ECM) production, and the migration of conjunctival and Tenon's capsule fibroblasts (Daniels et al. 1999; Jampel 1992; Lee et al. 1990; Occleston et al. 1997; Yamamoto et al. 1990). Together, these effects reduce the number of fibroblasts and attenuate postoperative scarring.

Despite MMC use, it is not uncommon for the effect of GFS to weaken or even disappear over time. In such cases, it has been reported that excessive postoperative wound‐healing in the conjunctival tissue causes fibrosis of the filtering bleb (Schlunck et al. 2016). MMC acts on fibroblasts to suppress excessive wound‐healing responses, but also exerts a paradoxical effect: MMC enhances the production of inflammatory cytokines such as IL‐8, MCP‐1, FGF2, and TGF‐β by various types of fibroblasts (Chen et al. 2006; Chou et al. 2007; Ho et al. 2014). These cytokines may exacerbate inflammation and ECM production, potentially compromising the surgical outcomes. In fact, elevated MCP‐1 levels in the aqueous humor are associated with poor prognosis after trabeculectomy (Inoue et al. 2014; Yu et al. 2021). These results suggest that a more comprehensive understanding of the effects of MMC on GFS may improve the treatment outcomes.

In this study, we comprehensively analyzed postoperative tissue samples to investigate the effects of MMC on GFS. We found that MMC promotes the expression of genes related to cellular senescence and inflammation. In vitro experiments and single‐cell RNA sequencing (scRNA‐Seq) analyses suggested that these changes were induced by MMC‐triggered senescence‐associated secretory phenotype (SASP)‐like alterations in the fibroblasts. Importantly, SASP‐like fibroblasts can be selectively eliminated by a specific senolytic agent, highlighting a novel therapeutic target that may improve surgical outcomes.

## 2 Methods

### 2.1 Animal Experiments

All animal experiments were approved by the Animal Care and Use Committee of Kumamoto University. Male and female Japanese white rabbits aged 12–14 weeks were subjected to trabeculectomy. The rabbits were purchased from KBT Oriental (Saga, Japan). Trabeculectomy without MMC was performed on 18 female rabbits to collect RNA for RNA‐sequencing (RNA‐Seq) analysis. In addition, six male animals underwent trabeculectomy with MMC for scRNA‐Seq analysis. For euthanasia, thiopental (100 mg/kg; Nipro, Osaka, Japan) was intravenously administered through the marginal ear vein.

### 2.2 Trabeculectomy

Trabeculectomy was performed as previously described, with minor modifications (Fujimoto et al. 2021; Watanabe‐Kitamura et al. 2021). Surgery was performed under anesthesia after intramuscular injection of ketamine hydrochloride (50 mg/kg; Daiichi Sankyo Propharma, Tokyo, Japan) and xylazine hydrochloride (10 mg/kg; Bayer HealthCare, Leverkusen, Germany). The conjunctiva was incised from the limbus, and a scleral flap was created, followed by treatment with a sponge soaked in MMC (0.4 mg/mL; Kyowa Kirin, Tokyo, Japan) for 3 min. When MMC was not used, phosphate‐buffered saline (PBS) was employed. The trabecular meshwork tissue under the scleral flap was removed, and peripheral iridectomy was performed. The scleral flap and conjunctiva were sutured with 10–0 nylon. To reduce inflammation and prevent infection, 0.4% rinderon (Shionogi Pharma Co. Ltd., Osaka, Japan) was administered subconjunctivally, and erythromycin lactobionate and colistin methanesulfonate ophthalmic ointment (Santen Pharmaceutical Co. Ltd., Osaka, Japan) was applied.

### 2.3 Intraocular Pressure Measurement

Intraocular pressure (IOP) was measured using a Tonovet device (Icare, Helsinki, Finland) when the rabbits were conscious. In rabbits that underwent trabeculectomy for RNA collection, IOP was measured the day before surgery and on Days 1, 7, and 14 postoperatively. Only rabbits that underwent sample collection 3 days after trabeculectomy were measured 3 days after surgery.

### 2.4 RNA Sequencing Analysis of Rabbit Bleb Conjunctiva

Previous sequence data on trabeculectomy with MMC samples (Watanabe‐Kitamura et al. 2021, GEO accession number: GSE156781) were used for comparative analysis with data on trabeculectomy without MMC. RNA was extracted from the bleb conjunctiva at 3 h, 3 days, and 14 days after trabeculectomy performed without MMC, as previously described (Fujimoto et al. 2021; Watanabe‐Kitamura et al. 2021) using TRIzol reagent, Phasemaker tubes, and the PureLink RNA Mini Kit (Thermo Fisher Scientific, Rockford, IL, USA). The collected RNA samples were verified, and RNA‐Seq was performed by Takara Bio (Shiga, Japan) as follows. For quality assessment of RNA samples, we used the Agilent 2200 TapeStation (Agilent Technologies, Santa Clara, CA, USA) or the Agilent 2100 Bioanalyzer (Agilent Technologies), and samples with RNA integrity ≥ 7 were used in further analyses. Sequencing libraries were prepared using the TruSeq Stranded mRNA Library Prep System (Illumina, San Diego, CA, USA) according to the manufacturer's instructions (TruSeq Standard mRNA Reference Guide v00). The Agilent 2100 Bioanalyzer was used to quality check the final libraries, and sequencing was performed on the NovaSeq 6000 platform (Illumina). The DRAGEN Bio‐IT Platform (Illumina, version 3.6.3) was used to map and calculate gene and transcript expression levels using both previously obtained sequence data (with MMC treatment) and newly obtained sequence data (without MMC treatment). The 150‐bp paired‐end reads were mapped to the rabbit genome sequence using OryCun2.0 (Ensembl). Expression levels are shown as raw and normalized counts (transcripts per million, TPM) for each gene and transcript. RNA‐Seq data were analyzed and visualized using RNAseqChef (Etoh and Nakao 2023). For the statistical analysis of RNA‐seq data, DESeq2 was used to perform normalization and extract differentially expressed genes (DEGs), and the false discovery rate (FDR) was adjusted using the Benjamini‐Hochberg method. The statistical significance level was an FDR ≤ 0.05 and an absolute log_2_ fold change (FC) ≥ 1. The hallmark gene set of the Molecular Signatures Database (MSigDB) was selected for the enrichment analysis.

### 2.5 Cell Culture and Treatment

Primary human conjunctival fibroblasts (HConFs) (cat. No. 6570, cell lot No. 5965, 20186, 20258, and 29536) were purchased from ScienCell Research Laboratories (Carlsbad, CA, USA). Cells were cultured in fibroblast medium supplemented with FBS, a fibroblast growth supplement, and antibiotics (penicillin/streptomycin solution; ScienCell Research Laboratories). HConFs were used at passage 6, except for experiments involving culture‐induced senescence. Appropriate morphology and expression of the fibroblast marker fibroblast‐specific protein 1 (cat. No. 27957; Abcam, Cambridge, UK) were confirmed in all cell strains as described previously (Futakuchi et al. 2016). HConFs were treated with MMC (Fujifilm Wako Pure Chemical, Osaka, Japan; concentration range: 0.001–0.3 mg/mL in PBS) or PBS for 5 min and washed with PBS. Then, the cells were cultured in low‐glucose Dulbecco's modified Eagle's medium (DMEM; Fujifilm Wako Pure Chemical) supplemented with 10% fetal bovine serum (FBS; HyClone, Cytiva, Tokyo, Japan) and an antibiotic–antimycotic (Thermo Fisher Scientific).

### 2.6 Cell Proliferation Assay

HConFs were seeded into 96‐well plates at a density of 3.0 × 10^3^ cells per well and treated with MMC at concentrations ranging from 0.003 to 0.3 mg/mL for 5 min. For the WST‐8 cell proliferation assay, we used the Cell Counting Kit‐8 (CCK‐8, Dojindo, Kumamoto, Japan), as described previously (Matsumura et al. 2020). WST‐8 assays were performed 3 h (Day 0) and 1, 3, 5, and 7 days after MMC treatment. At each time point, 10 μL of CCK‐8 solution was added to each well, and the cells were incubated at 37°C for 2 h. After incubation, the absorbance was measured at 450 nm using a microplate reader (Multiskan FC; Thermo Fisher Scientific).

### 2.7 Immunofluorescence Assay

Immunostaining was performed on HConFs 3 days after MMC treatment. HConFs were washed with PBS and fixed in 4% (v/v) paraformaldehyde in PBS for 15 min at room temperature, and then washed twice with PBS. For permeabilization, cells were incubated with 0.5% (v/v) Triton X‐100 in PBS for 12 min at room temperature and then blocked with serum buffer (10% FBS and 0.2 mg/mL sodium azide in PBS) at room temperature for 1 h. The cells were then incubated with anti‐γ‐H2AX antibody (1:400, cat. No. 9718; Cell Signaling Technology, Danvers, MA, USA) at 4°C overnight. After three washes with PBS, the cells were incubated with anti‐rabbit IgG secondary antibody Alexa Fluor 647 (1:400, cat. No. A21244; Thermo Fisher Scientific) and/or DAPI solution (dilution 1:1000, cat. No. D523; Dojindo Laboratories) at room temperature for 1 h. The cells were washed three times with PBS and observed under a fluorescence microscope (BZ‐X710; Keyence Japan, Osaka, Japan).

### 2.8 Senescence‐Associated β‐Galactosidase (SA‐β‐Gal) Staining

For SA‐β‐Gal staining, we used the Senescence‐associated β‐Galactosidase Staining Kit (Cell Signaling Technology) following the manufacturer's protocol. Briefly, HConFs on Day 3 post‐MMC treatment were fixed in 1× fixative solution for 10 min, washed twice with PBS, and incubated with the staining solutions for 24 h at 37°C. Senescent cells were observed using a fluorescence microscope (BZ‐X710; Keyence).

### 2.9 Western Blotting

Samples for Western blotting were collected at 3 h, 3 days, and 7 days after MMC treatment of HConFs. Western blotting was conducted as described previously with minor modifications (Matsumura et al. 2020; Watanabe‐Kitamura et al. 2021) using the following primary antibodies: anti‐GAPDH (1:10,000, cat. No. G8795; Sigma‐Aldrich, St. Louis, MO, USA), anti‐p21 (1:1000, cat. No. 2947S; Cell Signaling Technology), anti‐p16 (1:1000, cat. No. ab108349; Abcam), and anti‐caspase 3 (1:1000, cat. No. 14220S; Cell Signaling Technology). The secondary antibodies included horseradish peroxidase (HRP)‐linked anti‐rabbit antibody (1:2000, cat. No. 7074; Cell Signaling Technology) and anti‐mouse antibody (1:4000, cat. No. 7076; Cell Signaling Technology).

### 2.10 RNA‐Seq Analysis of HConFs After MMC Treatment

RNA was collected from HConFs 3 h, 3 days, and 14 days after 0.1 mg/mL MMC treatment and subjected to RNA‐Seq analysis. RNA was isolated from the cells using a NucleoSpin RNA kit (Takara Bio) according to the manufacturer's protocol. Sequencing libraries were synthesized using the TruSeq Stranded mRNA Library Prep System (Illumina) according to the manufacturer's instructions. Sequencing was performed using a NextSeq 500 Sequencer (Illumina), yielding 75‐bp single‐end reads that were mapped to the human genome sequence using hg38 and STAR. The expression levels are shown as raw and normalized counts (TPM) for each gene. RNA‐Seq data were analyzed using RNAseqChef (Etoh and Nakao 2023). For the statistical analysis of RNA‐seq data, DESeq2 was used to perform normalization and extract DEGs, and the FDR was adjusted using the Benjamini‐Hochberg method. The significance level in the statistical analysis was an FDR ≤ 0.05 and an absolute log_2_FC ≥ 1. The hallmark gene set of MSigDB was selected for enrichment analysis. SASP‐related factors were selected based on the report by Kumari and Jat (2021), and changes in their expression were visualized using a heatmap (Figure 4A).

### 2.11 Quantitative Real‐Time PCR (qPCR)

Cellular RNA samples for qPCR were isolated from HConFs at 3 h, 3 days, and 7 days after MMC treatment using the NucleoSpin RNA kit (Takara Bio) according to the manufacturer's protocol. RNA was reverse‐transcribed using the PrimeScript RT‐PCR kit (Takara Bio) and subjected to qPCR (TB Green Premix Ex Taq II; Takara Bio) using a StepOnePlus Real‐time PCR System (Thermo Fisher Scientific). All expression data were normalized to the *GAPDH* mRNA level. For PCR, we used the following primers: *CDKN1A* (forward, 5′‐CGA TGG AAC TTC GAC TTT GTC A‐3′; reverse, 5′‐GCA CAA GGG TAC AAG ACA GTG‐3′), *CDKN2A* (forward, 5′‐GAT CCA GGT GGG TAG AAG GTC‐3′; reverse, 5′‐CCC CTG CAA ACT TCG TCC T‐3′), *FGF2* (forward, 5′‐AGA AGA GCG ACC CTC ACA TCA‐3′; reverse, 5′‐CGG TTA GCA CAC ACT CCT TTG‐3′), *IL6* (forward, 5′‐ACT CAC CTC TTC AGA ACG AAT TG‐3′; reverse, 5′‐CCA TCT TTG GAA GGT TCA GGT TG‐3′), *IL10* (forward, 5′‐TCA AGG CGC ATG TGA ACT CC‐3′; reverse, 5′‐GAT GTC AAA CTC ACT CAT GGC T‐3′), *CCL2* (forward, 5′‐CAT GAA AGT CTC TGC CGC CC‐3′; reverse, 5′‐GGG CAT TGA TTG CAT CTG GCT G‐3′), *CCL7* (forward, 5′‐CCC TCA CCC TCC AAC ATG AA‐3′; reverse, 5′‐TGA AGT ATT AAT CCC AAC TGG CTG A‐3′), *CCL8* (forward, 5′‐TGC TGA AGC TCA CAC CCT TG‐3′; reverse, 5′‐TGG AAT GGA AAC TGA ATC TGG CTG‐3′), *CXCL8* (forward, 5′‐GTG GAG AAG TTT TTG AAG AGG GCT G‐3′; reverse, 5′‐ACT GGC ATC TTC ACT GAT TCT TGG‐3′), *TGFB2* (forward, 5′‐CCA TCC CGC CCA CTT TCT AC‐3′; reverse, 5′‐AGC TCA ATC CGT TGT TCA GGC‐3′), and *GAPDH* (forward, 5′‐GCA CCG TCA AGG CTG AGA AC‐3′; reverse, 5′‐TGG TGA AGA CGC CAG TGG A‐3′).

### 2.12 Multiplex Bead‐Based Immunoassay

The multiplex bead‐based immunoassay was conducted as previously reported (Futakuchi et al. 2017), with minor modifications. After treatment with MMC at 0.001, 0.01, and 0.1 mg/mL, HConFs were cultured in 10% FBS DMEM for 8 h, and the culture medium was then changed to FBS‐free DMEM. Culture supernatants were collected 24 h after medium exchange and stored at −20°C before analysis. The number of HConFs in each well was counted, and the concentration of each analyte was adjusted according to the cell number to measure the levels of secreted factors per cell. After thawing, the media were diluted twofold, and human magnetic Luminex screening assays (R&D Systems, Minneapolis, MN, USA) were performed according to the manufacturer's instructions to measure the following factors: FGF2, CCL2/MCP‐1, IL‐6, CXCL8/IL‐8, IL‐10, PDGF‐AA, and TGF‐β2. All data were acquired using the Luminex 200 System (Luminex) and analyzed using MasterPlex QT version 5.0 (Hitachi Solutions, Tokyo, Japan).

### 2.13 Single Cell RNA‐Seq (scRNA‐Seq) Analysis

Rabbit conjunctivae were dissociated using a modified version of a previous method (Naganuma et al. 2021) and subjected to scRNA‐Seq analysis. Briefly, one conjunctival tissue for each condition (non‐operated, 3 h, 3 days, and 14 days) was digested in collagenase solution for 10 min and treated with dissociation buffer for 10 min, followed by 0.25% trypsin/EDTA (200 μL) for 10 min, and 1 mL of Hank's balanced salt solution (HBSS)/10% FBS containing 10 μg/mL DNaseI and incubation at 37°C. After washing with PBS, cells were washed again with HBSS containing 2% FBS, 50 μg/mL DNase I, 1 mM CaCl_2_ (Fujifilm Wako Pure Chemical), and 0.035% NaHCO_3_ (Fujifilm Wako Pure Chemical). Finally, the cells were resuspended in 0.04% BSA/PBS, filtered through a 40 μm‐pore strainer (Corning, Corning, NY, USA), and evaluated in terms of cell number and viability (> 80%) using a Countess 3 automated cell counter (Thermo Fisher Scientific).

All libraries were prepared using the 10X Chromium system of the Chromium NextGEM Single Cell 3′ Kit v. 3.1, according to the manufacturer's protocol. Sequencing was performed on a NovaSeq X plus platform (first read, 28 bp; second read, 90 bp). Raw sequence data were processed using the count command of Cell Ranger (version 8.0.1; 10× Genomics) and mapped to a rabbit reference dataset (UM_NZW_1.0) to generate count tables of unique molecular identifiers (UMIs) for each gene per cell. The dataset was integrated using the cellranger aggr command. All subsequent analyses were performed in the R statistical programming language (version 4.4.0). The Seurat package version 5.0.3 (Hao et al. 2024) was used for quality control, data normalization, scaling, dimensionality reduction, clustering, and visualization. For quality control, cells that expressed ≤ 300 genes, ≤ 0.5% or ≥ 30% of mitochondrial genes, or a UMI number ≤ 600 were filtered out. The final dataset contained 23,587 genes and 14,859 cells. To correct for batch effects in the datasets, the RPCAIntegration method was used. Cluster segmentation was performed at a resolution of 0.4. Each cluster was manually annotated based on the expression of known marker genes. The uniform manifold approximation and projection for dimension reduction (UMAP) coordinates, Seurat cluster coordinates, and cluster‐specific markers obtained were exported as CSV files for confirmation analysis using Loupe Cell Browser software (10× Genomics, Pleasanton, CA, USA).

### 2.14 Cell–Cell Communication Analysis

The annotated Seurat object was used as the input for CellPhoneDB (v5), which was run separately for each time point. Fibroblasts were defined as a population that combined clusters #0, #3, #5, #6, and #14‐3. M1 and M2 macrophages were defined as clusters #7 and #1, respectively. We calculated significant ligand–receptor interactions (*p* value < 0.05) using the default parameters, except for an increase in the number of iterations to 10,000. To focus on cytokine‐related interactions, we extracted only the outputs annotated “Cytokine” from the CellPhoneDB database. We selected the 25 interactions with the highest significant mean values from the extracted interactions but used all interactions if there were fewer than 25. The resulting outputs were visualized as a chord diagram using the chorddiag package (version 0.1.3).

### 2.15 Effects of Senolytic Drugs

We evaluated ABT‐263, dasatinib plus quercetin, and BPTES as senolytic drugs against MMC‐induced cellular senescence in HConF. MMC treatment was performed the day after cell seeding, and senolytic drugs were added 7 days later. Senolytic drugs were added the day after cell seeding for cells not treated with MMC. Cell counts were performed immediately before the senolytic drug treatment and at 1, 2, and 3 days post‐treatment. For qPCR analysis, cells were collected 2 days after treatment with the senolytic drugs.

### 2.16 Statistics

For the data analysis, we used GraphPad Prism software (ver. 10.5.0; GraphPad Software, Boston, MA, USA). Statistical comparisons between two groups were performed using Student's *t*‐test, and for comparisons between three or more groups, we used one‐way analysis of variance (ANOVA) with Dunnett's post hoc test or Tukey–Kramer honestly significant difference (HSD) test. Differences were considered statistically significant at *p* < 0.05.

## 3 Results

### 3.1 MMC Enhances the Effects of GFS

To evaluate the effect of MMC in GFS, we assigned rabbits to either an MMC‐treated or a non‐treated group and performed trabeculectomy. Until postoperative Day 3, there was no significant difference in the IOP reduction between the two groups. However, on Days 7 and 14, the IOPs of the non‐treated group tended to return to the preoperative levels (ΔIOP –0.83 and −0.67 mmHg, respectively), whereas the MMC‐treated group maintained a sustained reduction in IOP (ΔIOP –7.33 and −7.39 mmHg, respectively) (Figure S1A). Additionally, the bleb morphology was preserved to 14 days postoperatively in only the MMC‐treated group (Figure S1B). These findings confirm that MMC prolongs IOP reduction and aids bleb preservation following trabeculectomy.

### 3.2 MMC Promotes the Expression of Genes Related to Cellular Senescence and Inflammatory Responses in Postoperative GFS Tissue

**FIGURE 1:**
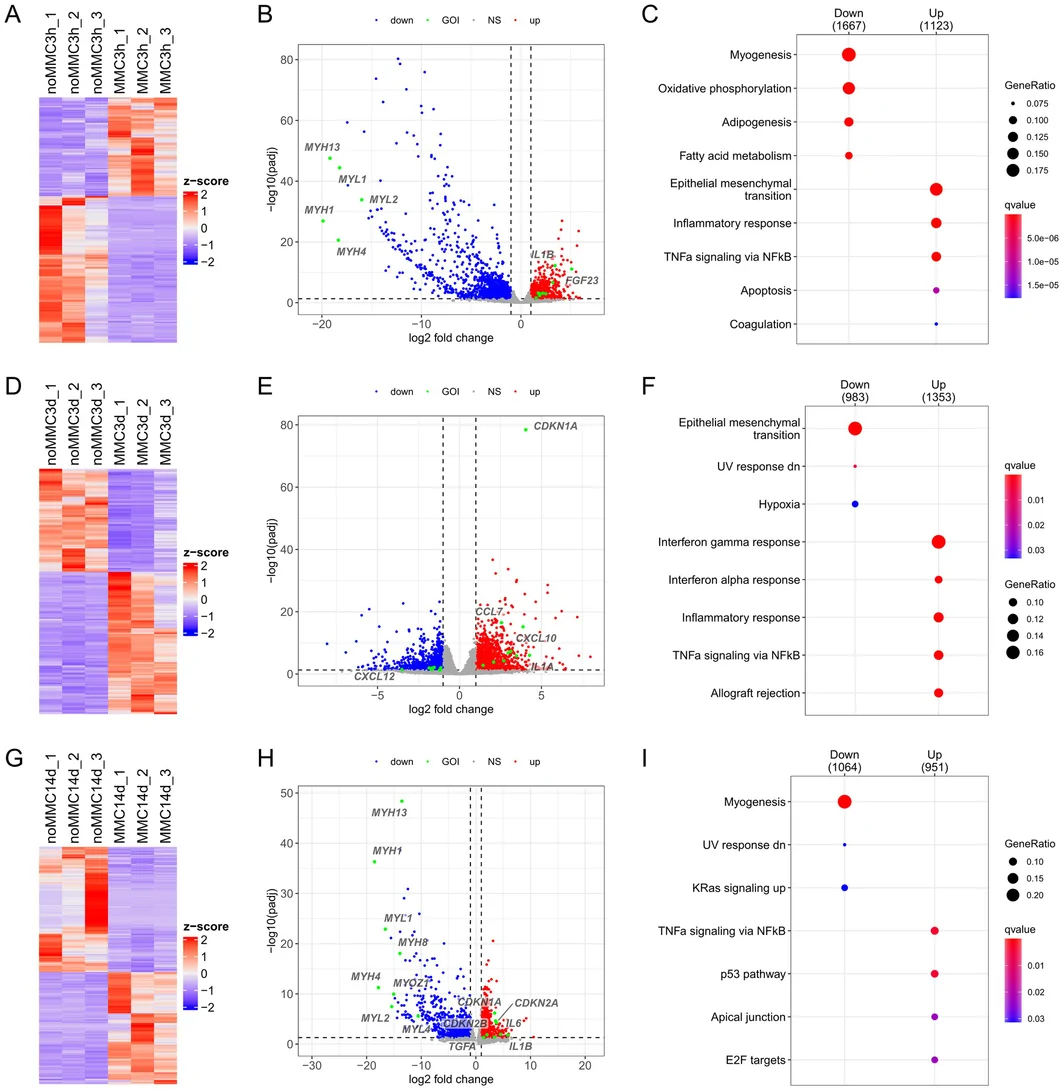
RNA expression in rabbit filtering bleb conjunctival tissue. Results of RNA‐Seq analysis of filtering bleb conjunctiva after trabeculectomy are shown. mRNAs expressed with or without MMC were compared 3 h (A–C), 3 days (D–F), and 14 days (G–I) after surgery. Genes exhibiting significant changes in expression are shown in the heatmaps (A, D, G) and volcano plots (B, E, H). Genes with significant changes were enriched using the MSigDB hallmark gene set (C, F, I).

To analyze the effects of MMC on postoperative conjunctival tissue, RNA‐Seq was performed on rabbit conjunctival tissues, including filtration blebs, following trabeculectomy with or without MMC. This revealed changes in the expression levels of 8553 genes in the MMC‐treated group and 7264 genes in the non‐treated group compared to the non‐operated conjunctival tissue (control, Figure S2). When comparing genes that significantly changed compared to the control at the same postoperative time point, the genes that changed differed considerably depending on whether MMC was used (Figure S3, Tables S1–S6). To examine the effects of MMC in more detail, we directly compared and analyzed the RNA‐Seq data from the MMC‐treated and non‐treated groups. Differences in gene expression were observed in 2790, 2336, and 2015 genes at 3 h, 3 days, and 14 days postoperatively, respectively (Figure 1, Tables S7–S9). Gene set enrichment analysis using the MSigDB hallmark gene set revealed that the genes upregulated at 3 h and 3 days postoperatively were significantly enriched in those related to inflammation, including the “inflammatory response”, “immune response”, and “interferon gamma response” (Figure 1C,F, Tables S10–S12). At postoperative Day 14, genes related to the p53 pathway, which is implicated in cellular senescence, were upregulated (Figure 1I, Table S13). These results indicate that MMC enhances the expression of genes involved in inflammation and cellular senescence in the bleb conjunctiva after GFS. In contrast, among the genes that showed a decrease, the most representative change was in ‘myogenesis,’ which includes myosin‐related genes such as MYH1, which showed significant changes at 3 h and 14 days postoperatively (Figure 1C,I, Tables S7, S9, S11, and S13).

### 3.3 MMC Induces Senescence‐Like Changes in Human Conjunctival Fibroblasts

To further investigate the effects of MMC on cellular senescence, we conducted experiments on cultured HConFs. We first continuously passaged HConFs and compared the cells at passages 6 and 17. Cells at passage 17 tended to be larger than those at passage 6 (Figure S4A). Western blotting showed increased expression of p16 and p21 (Figure S4B), both of which are associated with cellular senescence, confirming that passage induced senescence.

**FIGURE 2:**
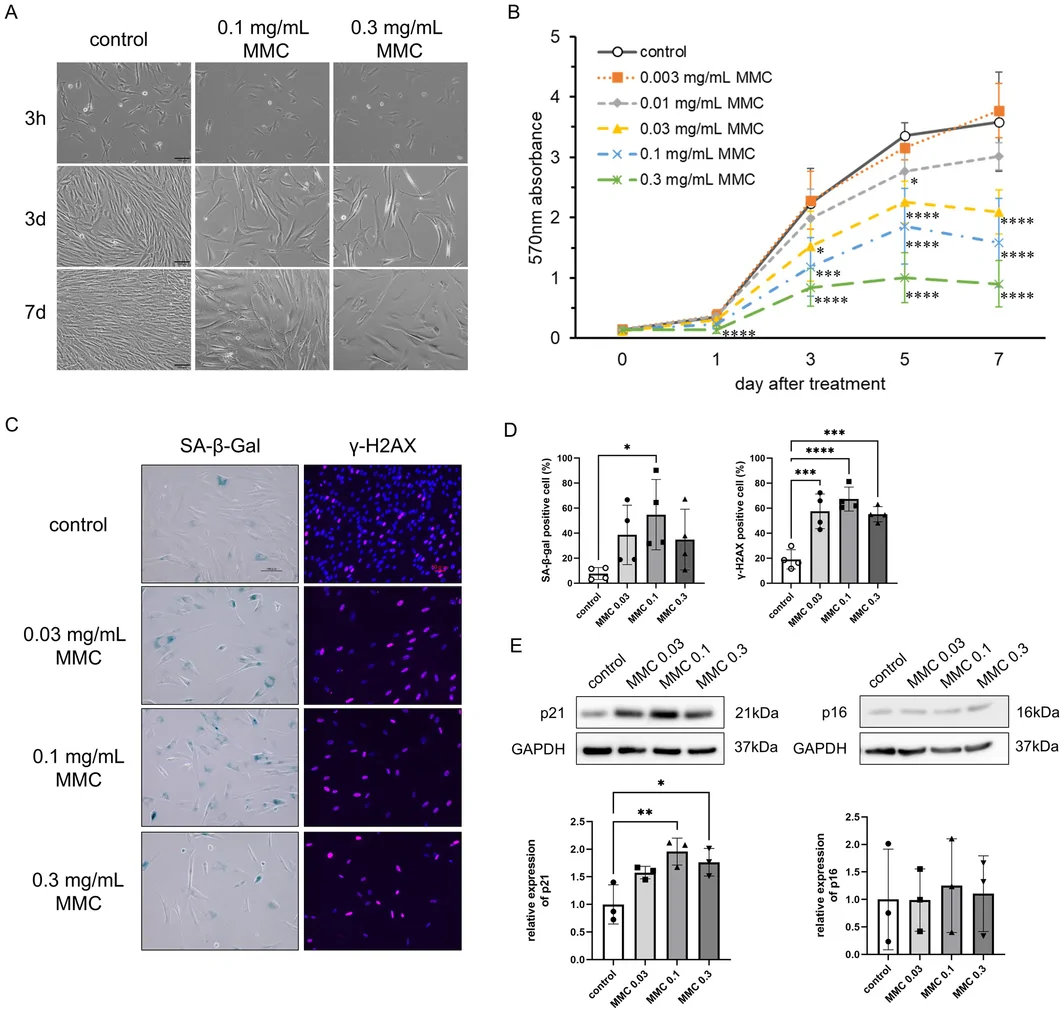
MMC induces senescence of human conjunctival fibroblasts (HConFs). (A) Phase‐contrast images of HConFs after MMC treatment are shown (scale bar: 100 μm). (B) Effect of MMC on HConF proliferation was assessed using the WST‐8 assay. (C) Images of SA‐β‐Gal and γ‐H2AX staining 3 days after MMC treatment are shown (scale bar: 100 μm). (D) Percentage of SA‐β‐Gal‐positive (left) and γ‐H2AX‐positive (right) HConFs 3 days after MMC treatment are shown. (E) p21 and p16 expression by HConFs after treatment with three concentrations of MMC (0.03, 0.1, and 0.3 mg/mL) for 3 days was analyzed using western blotting. Representative bands and their relative changes are shown. (B) *n* = 12, (D, E) *n* = 3, *p < 0.05, **p < 0.01, ***p < 0.001, ****p < 0.0001, Dunnett's test.

HConFs were treated with MMC to determine whether this induced senescence. Both a decrease in cell number and morphological enlargement were observed (Figure 2A). The WST‐8 assay confirmed the dose‐dependent suppression of cell proliferation (Figure 2B), and the number of cells positive for senescence markers, such as SA‐β‐Gal and γ‐H2AX, increased (Figure 2C,D). Western blot and qPCR analyses revealed increased p21 expression (encoded by *CDKN1A*) after MMC treatment (Figure 2E, Figure S4C,D,G). In contrast, p16 (*CDKN2A*) expression did not change significantly (Figure 2E, Figure S4E,F,H). These results suggest that HConFs that survived MMC treatment exhibited senescence‐like changes, although these were atypical.

### 3.4 MMC Promotes the Expression of SASP‐Related Factors in Conjunctival Fibroblasts

**FIGURE 3:**
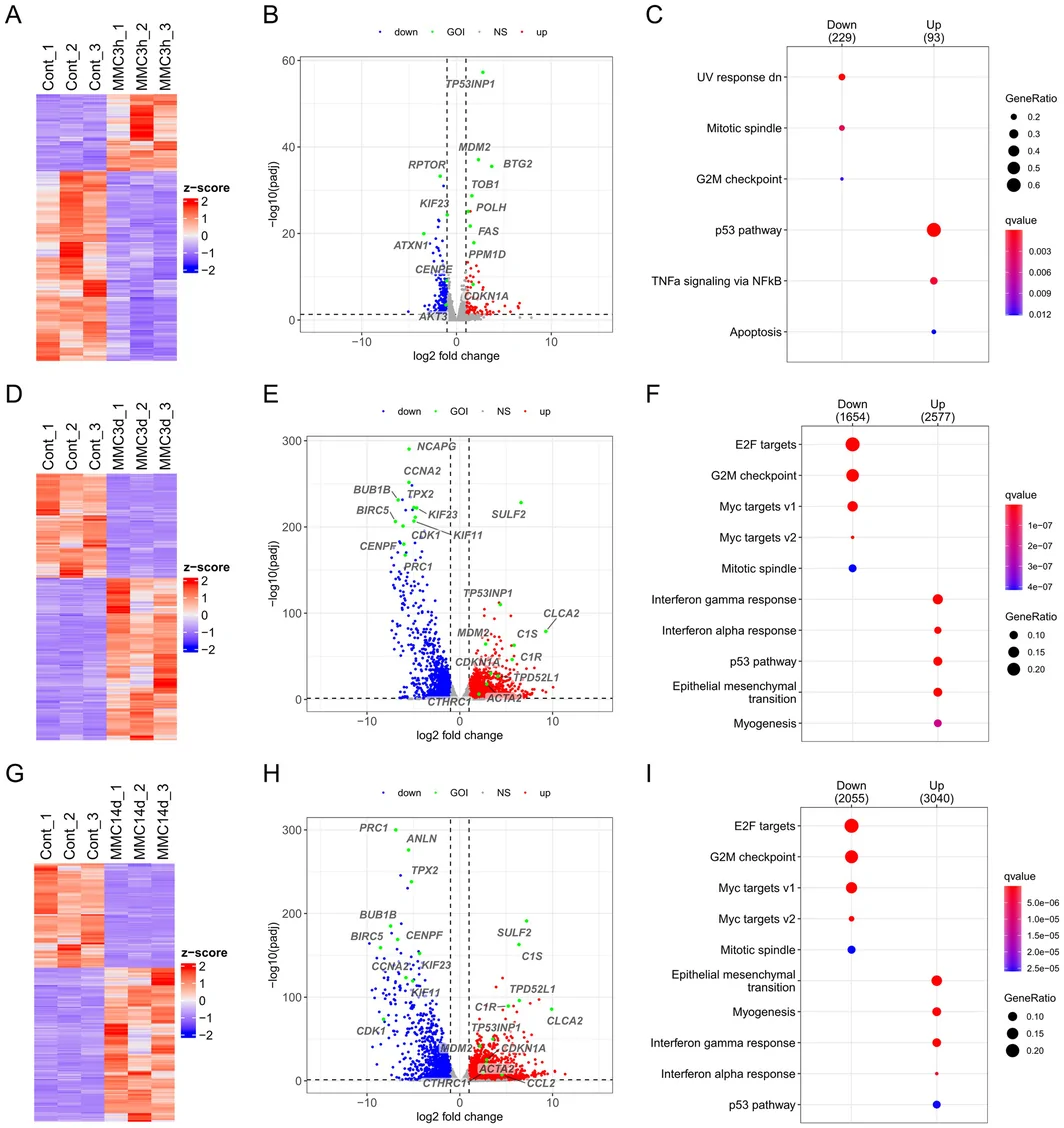
Comprehensive analysis of RNA expression changes after MMC treatment of HConFs. RNA‐Seq data of HConFs after MMC treatment are shown. Comparisons were made at 3 h (A–C), 3 days (D–F), and 14 days (G–I) after 0.1 mg/mL MMC treatment. Changes in gene expression are shown in the heatmaps (A, D, G) and volcano plots (B, E, H). Genes with significant changes in expression were subjected to enrichment analysis using the MSigDB hallmark gene set (C, F, I).

**FIGURE 4:**
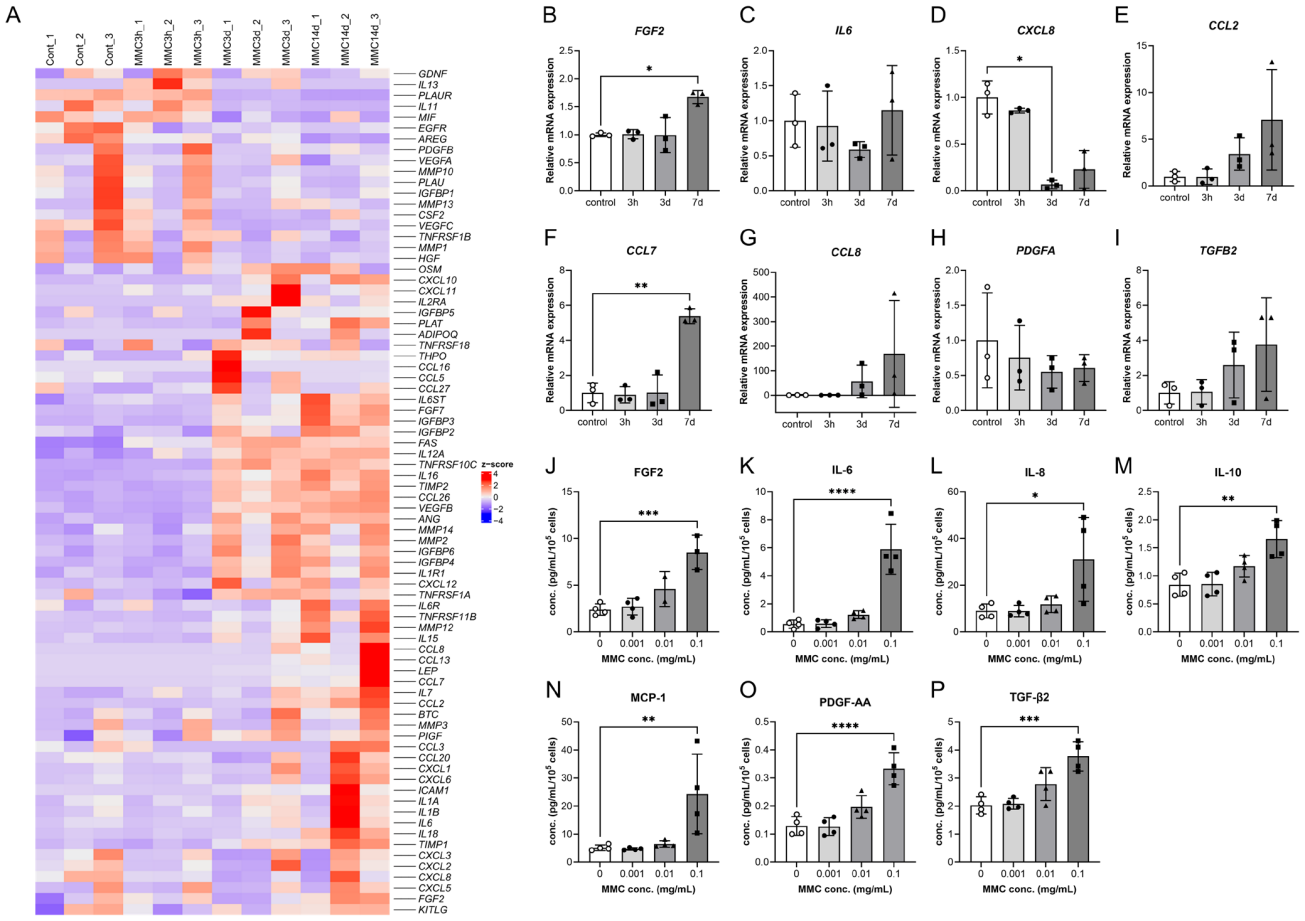
Changes in SASP‐related factors following MMC treatment of HConFs. (A) Heatmap showing changes in the expression levels of SASP‐related genes in HConF after 0.1 mg/mL MMC treatment. (B–I) Changes in the levels of mRNAs encoding SASP‐related factors after 0.1 mg/mL MMC treatment were examined using qPCR. Data are presented as mean ± standard deviation. (J–P) Changes in the expression levels of SASP‐related factors in the culture supernatant of HConFs after MMC treatment (0.001, 0.01, and 0.1 mg/mL) for 24 h. Data are presented as mean ± standard deviation. (B–I) *n* = 3, (J) *n* = 2–4, (K–P) *n* = 4, *p < 0.05, **p < 0.01, ***p < 0.001, ****p < 0.0001, Dunnett's test.

We performed RNA‐Seq to comprehensively analyze the gene expression changes induced by MMC in HConFs. Significant changes in the expression levels of 322, 4231, and 5095 genes were observed at 3 h, 3 days, and 14 days after MMC treatment, respectively (Figure 3, Tables S14–S16). Gene set enrichment analysis using the MSigDB hallmark gene set revealed upregulation of genes associated with “apoptosis” and “p53 pathway”, and downregulation of genes related to “G2M checkpoint” and “E2F targets” (Figure 3, Tables S17–S21). Thus, the enrichment analyses showed that MMC widely affected both apoptosis and cell‐cycle regulation, as expected. However, apoptosis induction by MMC was investigated using western blotting for caspase 3, but no expression of cleaved caspase 3 was observed 3 h after MMC treatment (Figure S4I). The heatmap illustrated the widespread upregulation of SASP‐related genes (Figure 4A). qPCR validated significant increases in *FGF2* and *CCL7* expression following MMC treatment. Although the expression levels of *CCL2*, *CCL8*, and *TGFB2* also tended to increase, the changes were not statistically significant (Figure 4B–I). No *IL10* mRNA was observed in the control, but *IL10* mRNA was clearly apparent 7 days after treatment with 0.1 mg/mL MMC (Figure S5). Furthermore, multiplex bead‐based assay of conditioned medium from MMC‐treated HConFs revealed significant increases in FGF‐2, IL‐6, IL‐8, IL‐10, MCP‐1, PDGF‐AA, and TGF‐β2 after treatment with 0.1 mg/mL MMC (Figure 4J–P). These results indicate that fibroblasts surviving after treatment with MMC exhibited an SASP‐like phenotype in vitro.

### 3.5 SASP Factors Are Produced by Fibroblasts After GFS With MMC

**FIGURE 5:**
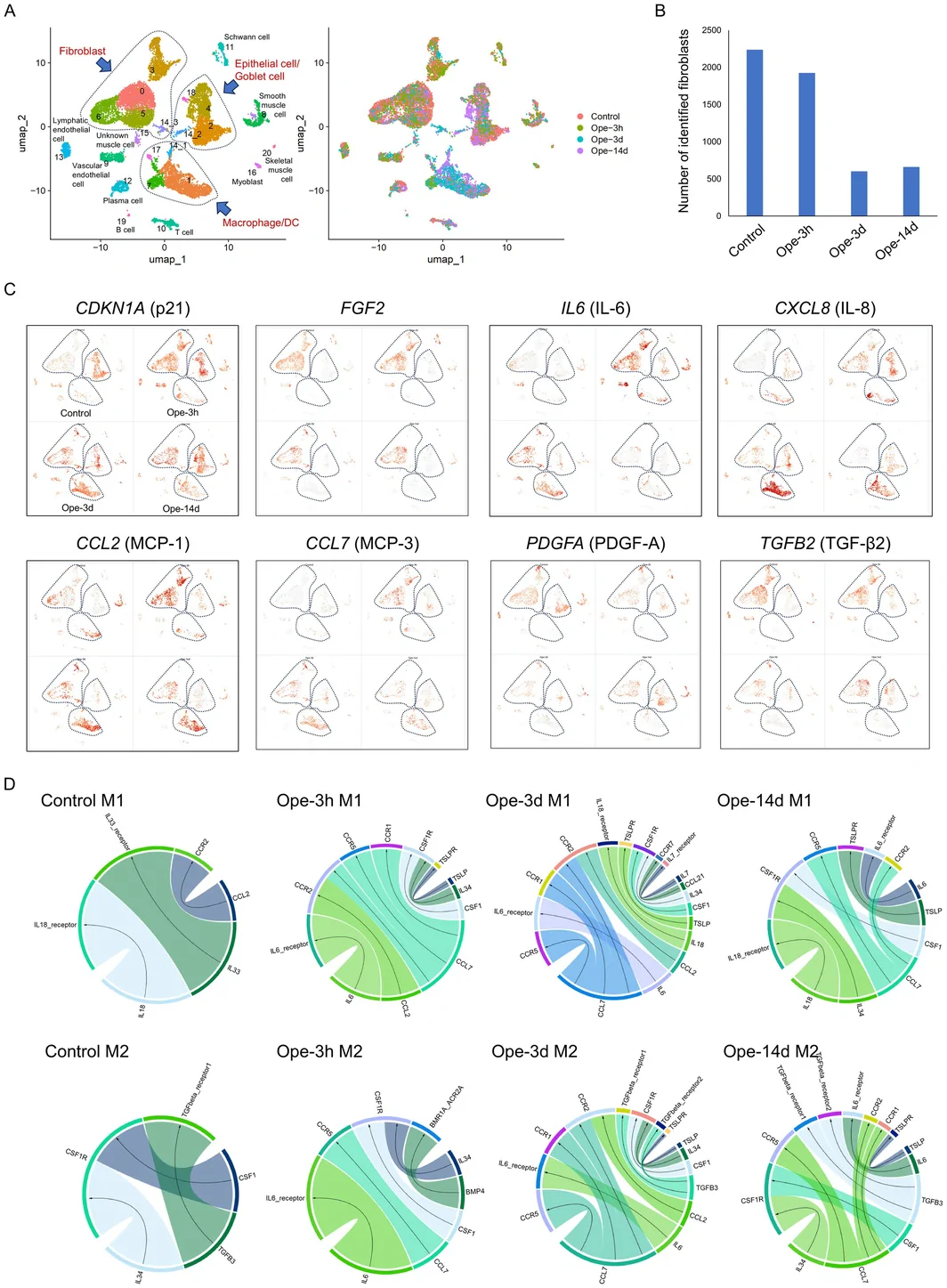
scRNA‐Seq analysis of rabbit filtering bleb conjunctival tissue. (A) UMAP‐based unbiased cluster classification of cells from rabbit GFS tissue is shown. In the left panel, clusters classified as fibroblasts, epithelial cells/goblet cells, and macrophages/dendritic cells (DCs), each surrounded by dotted lines, are represented in different colors. The details are provided in Figure S6. The experimental groups in the right panel differed by color. (B) Postoperative changes in the number of identified fibroblasts are shown. (C) Expression patterns of SASP‐related factors at each postoperative time point are displayed in feature plots. Each panel shows data for a single factor, with the upper left, upper right, lower left, and lower right panels containing data for the control and samples obtained 3 h (Ope‐3h), 3 days (Ope‐3d), and 14 days (Ope‐14d) after surgery, respectively. (D) The CellPhoneDB chord diagrams show the predicted ligand–target interactions of fibroblast‐derived SASP‐associated factors and macrophage receptors at each postoperative time point. The upper panels show interactions with M1 macrophages, and the lower panels show interactions with M2 macrophages.

To determine whether SASP‐like fibroblasts were involved in MMC‐induced inflammatory reactions, we subjected the bleb conjunctiva to scRNA‐Seq after GFS with MMC. We successfully sequenced 4349, 3683, 2588, and 2921 single cells from rabbit GFS control tissue (without surgery) and tissues obtained 3 h, 3 days, and 14 days after surgery, respectively. Unbiased clustering of single cells projected onto UMAP space identified 21 major clusters (Figure 5A and Figure S6). Of these, cluster #14, representative of proliferating cells, was subdivided into three clusters: #14‐1 (proliferating macrophages), #14‐2 (proliferating epithelial cells), and #14‐3 (proliferating fibroblasts). Fibroblasts were classified into five clusters: #0, #3, #5, #6, and #14‐3. On Days 3 and 14 after surgery, the number of fibroblasts was less than 30% that of the control group (Figure 5B), suggesting that MMC killed fibroblasts. SASP‐related factors, such as *CDKN1A*, *IL6*, *CXCL8*, *CCL2*, and *CCL7*, were upregulated in fibroblasts after surgery, although the induction of *IL6* and *CXCL8* did not persist to 14 days after surgery (Figure 5C). In contrast, the expression of *PDGFA* and *TGFB2*, both of which were upregulated by MMC treatment in vitro, did not change after surgery compared to the in vivo control. Thus, GFS with MMC induced the development of SASP‐like fibroblasts, the phenotype of which differed somewhat from that in vitro. These results suggest that various cell–cell interactions in distinct microenvironments after GFS with MMC application may affect the cellular reactions.

As SASP‐related factors attract pro‐inflammatory cells into various microenvironments, we used the CellPhoneDB method to investigate whether fibroblasts and macrophages interacted via SASP‐related factors after GFS (Efremova et al. 2020). Chord diagrams based on the mRNA expression levels of cytokines and known receptors revealed time‐dependent changes in the relationships between key fibroblast cytokines and their receptors on M1 and M2 macrophages (Figure 5D). The potential interactions became more diverse after surgery (compared to the control). This analysis suggested that CCL7 and its receptors, CCR1, CCR2, and CCR5, are likely the major molecules mediating communications between fibroblasts and both M1 and M2 macrophages after GFS.

### 3.6 Appropriate Senolytics Eliminate MMC‐Induced SASP‐Like Fibroblasts

**FIGURE 6:**
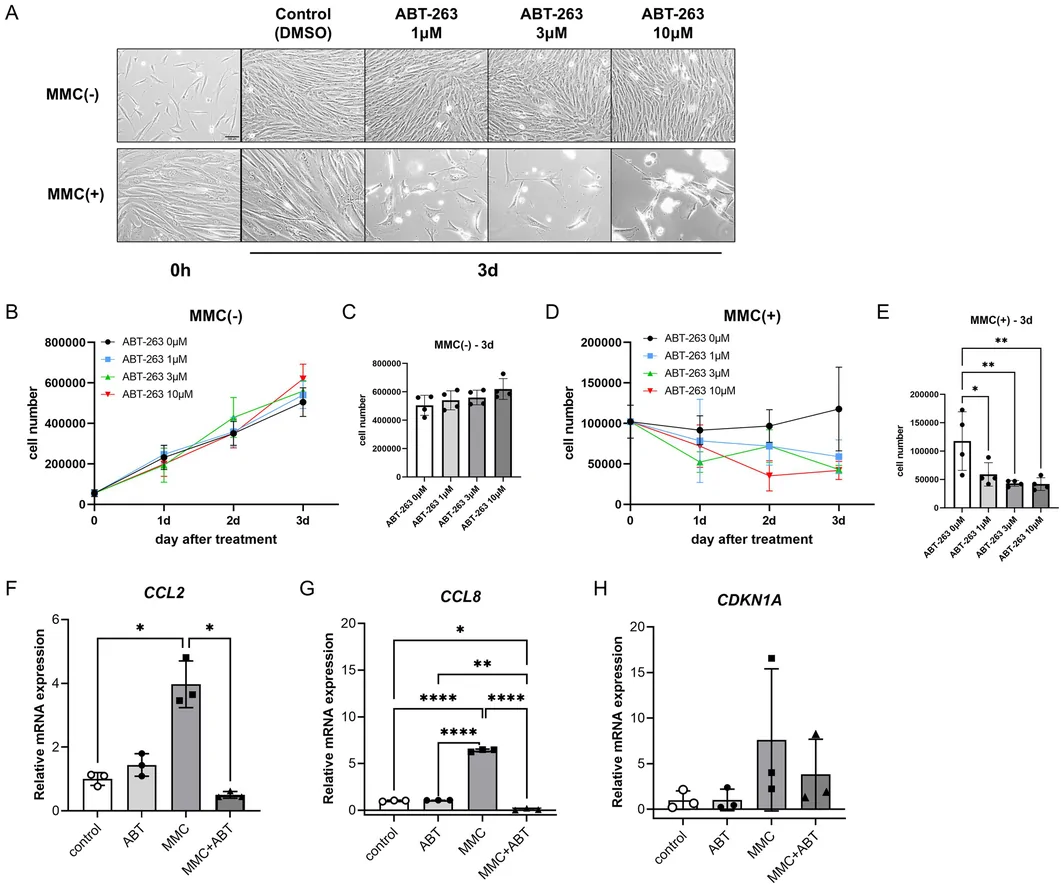
Effect of ABT‐263 on MMC‐induced cellular senescence. (A) Phase‐contrast images of HConFs before ABT‐263 treatment and after 3 days of ABT‐263 treatment are shown. The upper row shows cells not treated with MMC, and the lower row shows cells treated with MMC (scale bar: 100 μm). (B–E) Changes in cell numbers were measured 3 days after ABT‐263 treatment. The graphs show cells not treated with MMC (B, C) and cells treated with MMC (D, E). B and D show the changes over 3 days, and C and E show the cell numbers 3 days after treatment. *n* = 4, *p < 0.05, **p < 0.01, Dunnett's test. (F–H) ABT‐263‐induced changes in the levels of mRNAs encoding SASP factors, as revealed by real‐time PCR. Data are presented as mean ± standard deviation (*n* = 3). *p < 0.05, **p < 0.01, ****p < 0.0001, Tukey–Kramer HSD test.

We investigated whether fibroblasts exhibiting a SASP‐like phenotype after MMC treatment could be eliminated by senolytic agents. Treatment with a combination of dasatinib (a tyrosine kinase inhibitor) and quercetin was cytotoxic to both control and MMC‐treated cells (Figure S7A) but BPTES, a glutaminase GLS1 inhibitor, was not (Figure S7B), suggesting that neither senolytic was selectively cytotoxic to SASP‐like conjunctival fibroblasts. However, compared to the control, ABT‐263, a Bcl‐2 family inhibitor, specifically reduced fibroblast viability after MMC treatment (Figure 6A). ABT‐263 did not affect the cell counts of the control group (Figure 6A–C). In contrast, in the MMC‐treated group, ABT‐263 significantly reduced cell numbers to less than 40% that of the control after 3 days of treatment (Figure 6D,E). qPCR analysis revealed that expression of the SASP‐related genes *CCL2* and *CCL8* was significantly upregulated by MMC and significantly downregulated by ABT‐263 (Figure 6F,G). A similar trend was observed for *CDKN1A*, although statistical significance was not attained (Figure 6H). These findings suggest that conjunctival fibroblasts that acquire a SASP‐like phenotype in response to MMC treatment can be selectively eliminated by appropriate senolytic agents, in turn reducing the expression of SASP‐related genes. Thus, ABT‐263 may improve the surgical outcomes of GFS using MMC.

## 4 Discussion

MMC improves IOP control after GFS (Chen 1983; Kitazawa et al. 1991). MMC is now widely used to enhance the outcomes of trabeculectomy. In our rabbit GFS model, MMC supported postoperative IOP reduction and improved bleb preservation (Figure S1), likely attributable to a reduction in fibroblast numbers and suppression of fibroblast proliferation, as observed in our in vivo and in vitro experiments (Figures 2 and 5). However, paradoxically, MMC also promoted the secretion of pro‐inflammatory cytokines (Figures 4 and 5) that are known to enhance fibrosis and compromise surgical outcomes. Based on this, we hypothesized that key factors that might improve the postoperative results but were previously overlooked, given the marked benefits afforded by MMC, remained to be identified.

We observed a significant decrease in myosin‐related gene expression in the RNA‐Seq analysis of bleb tissue (Figure 1C,I, Tables S7, S9, S11, and S13). A detailed examination of the data revealed a trend toward increased expression of myosin‐related genes in postoperative tissues not treated with MMC compared to the controls (Table S1), suggesting that MMC treatment suppresses this upregulation. However, in our in vitro study using HConF, we did not observe a decrease in these genes following MMC treatment; instead, we found either no change or an increase in expression (Figure 3I, Tables S18 and S20), which differed from the results obtained from the tissue samples. One possible reason why this suppressive effect was not observed in vitro studies is that the baseline expression levels of myosin‐related genes were not high. Furthermore, scRNA‐Seq analysis of bleb tissue showed that myosin‐related gene expression remained at control levels even after surgery (Figure S8). Previous reports have indicated that short‐term MMC treatment of conjunctival fibroblasts inhibits collagen gel contraction in vitro (Occleston et al. 1994). These results suggest that MMC may influence the contractility of conjunctival fibroblasts, and this effect may be more pronounced in postoperative tissues.

Several studies have investigated the relationship between MMC use and cellular senescence. McKenna et al. reported that exposure of human pulmonary carcinoma cells to MMC at 0.01 or 0.02 μg/mL for 6 days induced senescence (McKenna et al. 2012). Lin et al., using human Tenon's fibroblasts, showed that treatment with 0.02 or 0.2 μM MMC for 48 h also triggered senescence (Lin et al. 2020). In both studies, MMC was used over the long term at relatively low concentrations. Here, we investigated the effects of short‐term (5 min) use of high‐concentration MMC (0.1 mg/mL) on HConFs, mirroring MMC use during GFS. MMC induced senescence‐like changes in surviving HConFs, including SA‐β‐gal positivity, γ‐H2AX accumulation, and increased p21 expression (Figure 3). These MMC‐treated cells secreted SASP factors, consistent with previous studies on mesenchymal stem cells (Ratushnyy et al. 2024). Moreover, using scRNA‐Seq, we demonstrated for the first time that conjunctival fibroblasts exhibited SASP‐like features in vivo following GFS with MMC, although the number of fibroblasts was reduced to less than 30% that of the control group (Figure 5). These findings suggest that SASP‐like fibroblasts that survive MMC treatment may contribute to bleb fibrosis after GFS via the secretion of pro‐inflammatory cytokines.

The importance of transient cellular senescence in the wound‐healing process has been supported by multiple studies, indicating that its presence at wound sites contributes to tissue regeneration, inflammation regulation, and fibrosis suppression. For example, transiently senescent cells secrete inflammatory cytokines and growth factors through the SASP, thereby promoting immune cell recruitment, angiogenesis, and cellular proliferation, all of which support normal and appropriate wound repair (Pils et al. 2021; Wilkinson and Hardman 2022). Consistent with these findings, the present study demonstrated a transient increase in SASP‐related factors during the early postoperative period in non‐MMC‐treated GFS eyes. In contrast, MMC‐treated GFS eyes exhibited an early postoperative increase and sustained elevation of SASP‐related factors, suggesting that MMC induces chronic cellular senescence.

CCL7, also called monocyte chemotactic protein‐3 (MCP‐3), is structurally similar to CCL2 (monocyte chemotactic protein‐1, MCP‐1) and acts through receptors including CCR1, CCR2, CCR3, and CCR5 (Cheng et al. 2014). CCL7 recruits monocytes to sites of tissue injury and contributes to local inflammatory responses. To the best of our knowledge, the only glaucoma‐related report to date describes elevated levels of CCL7 in the aqueous humor following acute angle‐closure glaucoma (Gao et al. 2016). In the present study, we report for the first time that CCL7 is produced by conjunctival fibroblasts following MMC treatment and conjunctival tissue after GFS with MMC. Moreover, this study suggested that CCL7 and its receptors are the major molecules mediating communication between fibroblasts and both M1 and M2 macrophages after GFS (Figure 5). As CCL7 promotes fibrosis of various organs including the skin, lung, and kidney (Gonzalez et al. 2013; Huang et al. 2024; Ong et al. 2003), CCL7 may be one of the key SASP factors involved in inflammatory cell recruitment and bleb fibrosis. Furthermore, this study also showed an increase in chemokines such as CCL2 and CCL8 following MMC treatment, in addition to CCL7. CCL2 and CCL8 are known to play significant roles in macrophage migration, activation, and the promotion of inflammation via the CCR2 receptor (Huang et al. 2020; Yen et al. 1997). While there are numerous reports regarding CCL2, we have previously reported it as a risk factor for glaucoma filtration surgery (Inoue et al. 2014). Furthermore, we recently reported using in vivo imaging in mice that CCL2 increases macrophage migration and tissue accumulation in the conjunctival tissue (Nakamura et al. 2026). The increase in these chemokines after GFS may act as a factor that promotes the aging environment, indirectly contributing to the maintenance and enhancement of senescent cellular colonies. However, further investigation is required.

Senescent cells develop resistance to death via activation of anti‐apoptotic pathways. Based on this, senolytic agents have been designed to preferentially eliminate senescent cells by targeting such survival mechanisms (Chaib et al. 2022). Although various senolytics are available, efficacy varies by the cell type and the mode of senescence induction (Power et al. 2023). We found that dasatinib plus quercetin and the GLS1 inhibitor BPTES were not selectively cytotoxic to MMC‐treated cells (Figure S7). In contrast, ABT‐263 exhibited a specific cytotoxic effect (Figure 6). ABT‐263 is an inhibitor of the anti‐apoptotic Bcl‐2 family proteins and has been reported to selectively eliminate senescent cells in cultures of HUVECs, IMR90 human lung fibroblasts, and murine embryonic fibroblasts (Zhu et al. 2016). In vivo, ABT‐263 reduced eyelid fibrosis in a mouse model of chronic graft‐versus‐host disease (Sato et al. 2024) and suppressed immune rejection and inflammatory cytokine production in a mouse model of corneal transplantation (Chi et al. 2025). In the present study, we found that ABT‐263 selectively eliminated SASP‐like conjunctival fibroblasts after MMC treatment and suppressed the expression of SASP‐related genes. These findings suggest that ABT‐263 may serve as a promising therapeutic strategy following GFS. Furthermore, our study is significant in that it is the first to identify the potential utility of senolytics, which may enhance the efficacy of GFS. Further studies are warranted to evaluate the safety and effectiveness of this strategy.

One limitation of this study is the lack of data from surgical tissues obtained in real clinical settings. Also, possible between‐species differences and the potential effects of topical antiglaucoma medications were not considered. Finally, the efficacy of ABT‐263 has not yet been evaluated in vivo, and further investigation is warranted to determine its effects on the filtering bleb.

Our findings demonstrate that MMC induces SASP‐like changes in fibroblasts, leading to enhanced expression of senescence‐ and inflammation‐related genes. The fact that a senolytic agent selectively eliminated these fibroblasts implies a promising therapeutic approach that might mitigate adverse effects associated with MMC treatment and improve postoperative outcomes. Targeting SASP‐like fibroblasts may be a novel strategy to enhance tissue healing and surgical success.

## Author Contributions

T.I. and T.F. conceptualized the study. A.K., T.F., S.I., and F.W.‐K. conducted the animal experiments. A.K., T.F., S.I., M.I., and U.T.‐K. performed the cell assays and obtained the data. T.F., S.I., and N.F. analyzed and visualized the RNA‐Seq data. S.I., M.I., S.T., and N.F. prepared the scRNA‐Seq samples. K‐.I.Y., T.K., D.K., and T.I. analyzed and visualized the scRNA‐Seq data. T.K. and M.N. proposed and reviewed the methods for RNA‐Seq and scRNA‐Seq data analysis. A.K., T.F., and T.I. created the figures. A.K., T.F., and T.I. wrote the original draft. All authors reviewed and edited the manuscript. T.F. and T.I. finalized the manuscript. T.I. supervised the study.

## Funding

This study was supported by the Inter‐University Research Network for High Depth Omics, IMEG, Kumamoto University, a MEXT grant (“Promotion of Development of a Joint Usage/Research System Project: Coalition of Universities for Research Excellence Program CURE”; grant no. JPMXP1323015486), and JSPS KAKENHI grants nos. JP21K09703 (T.I.) and JP23K15909 (F.W.‐K.).

## Ethics Statement

All animal experiments were approved by the Animal Care and Use Committee of Kumamoto University.

## Conflicts of Interest

The authors declare no conflicts of interest.

## Supporting information


**Data S1:** acel70680‐sup‐0001‐DataS1.xlsx.

**Figure S1:** Intraocular pressure and bleb shape in a rabbit trabeculectomy model. Changes in postoperative intraocular pressure (IOP) (A) and bleb shape (B) with or without MMC during trabeculectomy are shown. IOP was compared between the MMC‐treated (MMC) and non‐treated (noMMC) groups. *n* = 6 (*n* = 5 for only Day 7 in the MMC group), ***p < 0.001, Student's *t*‐test.
**Figure S2:** RNAs expressed by rabbit filtering bleb conjunctival tissue. The results of RNA‐Seq analysis of the conjunctiva of filtering blebs developing after trabeculectomy are shown. The mRNA expression patterns were compared with those of the controls. Changes in mRNA expression levels in the conjunctiva, including the bleb, after trabeculectomy with (A) and without (B) MMC are shown in the heatmap.
**Figure S3:** Venn diagram comparing genes altered in bleb conjunctiva after GFS with and without MMC treatment. Venn diagrams were used to compare the number of genes showing significant changes relative to the control (non‐operated eye) based on the presence or absence of MMC treatment. The figures display the results for 3 h (A, B), 3 days (C, D), and 14 days (E, F) post‐surgery. The left panels (A, C, E) show the number of upregulated genes, and the right panels (B, D, F) show the number of downregulated genes.
**Figure S4:** Cellular senescence of HConFs induced by passaging and MMC C. (A) Phase‐contrast images of HConFs at passages 6 (P6) and 17 (P17) are shown (scale bar: 100 μm). (B) Western blotting of p16 and p21 in HConFs at P6 and P17 is shown. (C–F) p21 and p16 expression levels in HConFs treated with three different concentrations of MMC (0.03, 0.1, and 0.3 mg/mL) for 3 h and 7 days, as revealed by western blotting; representative bands (C, E) and relative changes (D, F) are shown. (G, H) Changes in the levels of *CDKN1A* (E) and *CDKN2A* (F) mRNAs, as revealed by qPCR 3 h, 3 days, and 7 days after treatment with 0.1 mg/mL MMC. (I) Caspase 3 expression levels in HConFs treated with three different concentrations of MMC (0.03, 0.1, and 0.3 mg/mL) for 3 h, as revealed by Western blotting. Data are presented as mean ± standard deviation (*n* = 3). *p < 0.05, **p < 0.01, Dunnett test.
**Figure S5:** MMC‐induced *IL10* expression in HConFs. The levels of mRNA encoding *IL*10 in HConFs were evaluated using q‐PCR. Electrophoresis results of the PCR products are shown. Although *IL10* mRNA was absent in the control (sample 1), *IL10* mRNA expression was apparent 7 days after MMC treatment (sample 2). *GAPDH* mRNA, an endogenous control, was expressed at similar levels in both samples.
**Figure S6:** Cell clustering based on gene expression patterns of rabbit conjunctival tissue as revealed by scRNA‐Seq. The expression levels of various marker genes in each cell cluster of rabbit conjunctival tissue are displayed in violin (left) and dot plots (right).
**Figure S7:** Effects of senolytics on MMC‐induced cellular senescence. Phase‐contrast images of HConFs before culture and after 3 days of culture with dasatinib + quercetin (D + Q) (A) or BPTES (B) are shown. The upper row shows cells without MMC treatment, and the lower row shows cells treated with MMC (scale bar: 100 μm).
**Figure S8:** Changes in the expression of myosin‐related genes in scRNA‐Seq analysis. The expression patterns of myosin‐related genes at each postoperative time point are displayed in feature plots. Each panel shows data for a single factor, with the upper left, upper right, lower left, and lower right panels containing data for the control and samples obtained 3 h (Ope‐3 h), 3 days (Ope‐3d), and 14 days (Ope‐14d) after surgery, respectively. As shown in Figure 5A, the areas enclosed by dotted lines represent clusters of fibroblasts, epithelial cells/goblet cells, and macrophages/dendritic cells at the top left, top right, and bottom, respectively.
**Table S1:** Top 30 of upregulated genes after 3 h versus control.
**Table S2:** Top 30 of downregulated genes after 3 h versus control.
**Table S3:** Top 30 of upregulated genes after 3 days versus control.
**Table S4:** Top 30 of downregulated genes after 3 days versus control.
**Table S5:** Top 30 of upregulated genes after 14 days versus control.
**Table S6:** Top 30 of downregulated genes after 14 days versus control.
**Table S7:** Top 30 of significantly changed genes at 3 h after GFS—Comparison with GFS without MMC.
**Table S8:** Top 30 of significantly changed gene at 3d after GFS—Comparison with GFS without MMC.
**Table S9:** Top 30 of significantly changed genes at 14d after GFS—Comparison with GFS without MMC.
**Table S10:** Enrichment analysis using MSigDB Hallmark at 3 h‐up.
**Table S11:** Enrichment analysis using MSigDB Hallmark at 3 h‐down.
**Table S12:** Enrichment analysis using MSigDB Hallmark at 3 days.
**Table S13:** Enrichment analysis using MSigDB Hallmark at 14 days.
**Table S14:** Top 30 of significantly changed genes at 3 h after MMC treatment in HConFs.
**Table S15:** Top 30 of significantly changed genes at 3d after MMC treatment in HConFs.
**Table S16:** Top 30 of significantly changed genes at 14d after MMC treatment in HConFs.
**Table S17:** Enrichment analysis of MMC treated HConFs using MSigDB Hallmark at 3 h.
**Table S18:** Enrichment analysis of MMC treated HConFs using MSigDB Hallmark at 3d‐up.
**Table S19:** Enrichment analysis of MMC treated HConFs using MSigDB Hallmark at 3d‐down.
**Table S20:** Enrichment analysis of MMC treated HConFs using MSigDB Hallmark at 14d‐up.
**Table S21:** Enrichment analysis of MMC treated HConFs using MSigDB Hallmark at 14d‐down.

## Data Availability

The data that support the findings of this study will be available upon acceptance in NCBI SRA database at https://www.ncbi.nlm.nih.gov/sra, reference number GSE306050, GSE306051, and GSE307308.
