# Supplementary material for: Surviving Fibroblasts After Treatment With Mitomycin C Exhibit SASP‐Like Phenotype After Glaucoma Filtration Surgery
Source: Aging Cell. 2026 Aug 20;25(9):e70680. doi: 10.1111/acel.70680 (PMC13494357; doi:10.1111/acel.70680)
Supplement: Supplementary file 1 [file ACEL-25-e70680-s002.docx]

**Supplemental Figure S1. Intraocular pressure and bleb shape in a rabbit trabeculectomy model.**

Changes in postoperative intraocular pressure (IOP) (A) and bleb shape (B) with or without MMC during trabeculectomy are shown. IOP was compared between the MMC-treated (MMC) and non-treated (noMMC) groups. n = 6 (n = 5 for only day 7 in the MMC group), *** P < 0.001, Student’s t-test.

**Supplemental Figure S2. RNAs expressed by rabbit filtering bleb conjunctival tissue.**

The results of RNA-Seq analysis of the conjunctiva of filtering blebs developing after trabeculectomy are shown. The mRNA expression patterns were compared with those of the controls. Changes in mRNA expression levels in the conjunctiva, including the bleb, after trabeculectomy with (A) and without (B) MMC are shown in the heatmap.

**Supplemental Figure S3. Venn diagram comparing genes altered in bleb conjunctiva after GFS with and without MMC treatment.**

Venn diagrams were used to compare the number of genes showing significant changes relative to the control (non-operated eye) based on the presence or absence of MMC treatment. The figures display the results for 3 h (A, B), 3 d (C, D), and 14 d (E, F) post-surgery. The left panels (A, C, E) show the number of upregulated genes, and the right panels (B, D, F) show the number of downregulated genes.

**Supplemental Figure S4. Cellular senescence of HConFs induced by passaging and MMC C.**

(A) Phase-contrast images of HConFs at passages 6 (P6) and 17 (P17) are shown (scale bar: 100 µm). (B) Western blotting of p16 and p21 in HConFs at P6 and P17 is shown. (C-F) p21 and p16 expression levels in HConFs treated with three different concentrations of MMC (0.03, 0.1, and 0.3 mg/mL) for 3 h and 7 d, as revealed by western blotting; representative bands (C, E) and relative changes (D, F) are shown. (G, H) Changes in the levels of *CDKN1A* (E) and *CDKN2A* (F) mRNAs, as revealed by qPCR 3 h, 3 d, and 7 d after treatment with 0.1 mg/mL MMC. (I) Caspase 3 expression levels in HConFs treated with three different concentrations of MMC (0.03, 0.1, and 0.3 mg/mL) for 3 h, as revealed by western blotting. Data are presented as mean ± standard deviation (n = 3). *P < 0.05, **P < 0.01, Dunnett test.

**Supplemental Figure S5. MMC-induced *IL10* expression in HConFs.**

The levels of mRNA encoding *IL*10 in HConFs were evaluated using q-PCR. Electrophoresis results of the PCR products are shown. Although *IL10* mRNA was absent in the control (sample 1), *IL10* mRNA expression was apparent 7 d after MMC treatment (sample 2). *GAPDH* mRNA, an endogenous control, was expressed at similar levels in both samples.

**Supplemental Figure S6. Cell clustering based on gene expression patterns of rabbit conjunctival tissue as revealed by scRNA-Seq.**

The expression levels of various marker genes in each cell cluster of rabbit conjunctival tissue are displayed in violin (left) and dot plots (right).

**Supplemental Figure S7. Effects of senolytics on MMC-induced cellular senescence.**

Phase-contrast images of HConFs before culture and after 3 d of culture with dasatinib + quercetin (D + Q) (A) or BPTES (B) are shown. The upper row shows cells without MMC treatment, and the lower row shows cells treated with MMC (scale bar: 100 μm).

**Supplemental Figure S8. Changes in the expression of myosin-related genes in scRNA-Seq analysis.**

The expression patterns of myosin-related genes at each postoperative time point are displayed in feature plots. Each panel shows data for a single factor, with the upper left, upper right, lower left, and lower right panels containing data for the control and samples obtained 3 h (Ope-3h), 3 d (Ope-3d), and 14 d (Ope-14d) after surgery, respectively. As shown in Figure 5A, the areas enclosed by dotted lines represent clusters of fibroblasts, epithelial cells/goblet cells, and macrophages/dendritic cells at the top left, top right, and bottom, respectively.

**Supplemental Table 1 Top 30 of upregulated genes after 3h vs control**

| noMMC |  |  | MMC |  |  |
| --- | --- | --- | --- | --- | --- |
| Gene ID | SYMBOL | Fold Change | Gene ID | SYMBOL | Fold Change |
| ENSOCUG00000006159 | MYH15 | 12978.98 | ENSOCUG00000005666 | DHRS9 | 5835.19 |
| ENSOCUG00000011143 | FOSL1 | 1413.53 | ENSOCUG00000027471 | IL6 | 4062.42 |
| ENSOCUG00000005666 | DHRS9 | 708.77 | ENSOCUG00000014282 | IL20 | 3345.84 |
| ENSOCUG00000007035 | OSTN | 621.64 | ENSOCUG00000006660 | IL36G | 1829.86 |
| ENSOCUG00000014282 | IL20 | 533.50 | ENSOCUG00000007233 | IL23A | 1172.32 |
| ENSOCUG00000027471 | IL6 | 375.63 | ENSOCUG00000011835 | CXCL8 | 870.69 |
| ENSOCUG00000021775 | MMP10 | 347.41 | ENSOCUG00000001813 | ARG1 | 669.89 |
| ENSOCUG00000006901 | ANKRD1 | 341.45 | ENSOCUG00000008771 | IL1A | 571.46 |
| ENSOCUG00000001813 | ARG1 | 330.81 | ENSOCUG00000031088 | - | 516.88 |
| ENSOCUG00000011179 | TNMD | 328.25 | ENSOCUG00000011143 | FOSL1 | 443.60 |
| ENSOCUG00000011835 | CXCL8 | 325.80 | ENSOCUG00000021710 | CCL2 | 442.29 |
| ENSOCUG00000006831 | GNG4 | 239.85 | ENSOCUG00000001372 | ABCB11 | 437.49 |
| ENSOCUG00000029337 | MMP3 | 218.82 | ENSOCUG00000010142 | PTX3 | 372.48 |
| ENSOCUG00000031726 | - | 187.20 | ENSOCUG00000016068 | SERPINE1 | 356.12 |
| ENSOCUG00000022761 | IL3RA | 180.41 | ENSOCUG00000029337 | MMP3 | 333.12 |
| ENSOCUG00000000580 | - | 161.95 | ENSOCUG00000004935 | CYP24A1 | 324.41 |
| ENSOCUG00000031431 | - | 117.49 | ENSOCUG00000034927 | CCL7 | 318.95 |
| ENSOCUG00000008632 | S100A9 | 115.89 | ENSOCUG00000010336 | NTS | 309.12 |
| ENSOCUG00000030930 | - | 111.94 | ENSOCUG00000021580 | FGF23 | 298.74 |
| ENSOCUG00000027187 | PRG4 | 111.06 | ENSOCUG00000037423 | KRT6C | 294.97 |
| ENSOCUG00000023449 | HMGB2 | 106.76 | ENSOCUG00000031131 | - | 276.29 |
| ENSOCUG00000008606 | FPR2 | 106.70 | ENSOCUG00000021775 | MMP10 | 240.53 |
| ENSOCUG00000023958 | IL1B | 102.51 | ENSOCUG00000023958 | IL1B | 227.76 |
| ENSOCUG00000007816 | C1orf162 | 93.75 | ENSOCUG00000008606 | FPR2 | 213.59 |
| ENSOCUG00000009566 | S100A8 | 91.72 | ENSOCUG00000013688 | TNFAIP6 | 205.83 |
| ENSOCUG00000034944 | - | 89.97 | ENSOCUG00000006831 | GNG4 | 194.62 |
| ENSOCUG00000022235 | CEBPE | 89.02 | ENSOCUG00000024540 | - | 194.23 |
| ENSOCUG00000022646 | S100A12 | 87.56 | ENSOCUG00000027272 | GPR3 | 182.45 |
| ENSOCUG00000016661 | DRC7 | 81.69 | ENSOCUG00000014998 | IL36A | 173.41 |
| ENSOCUG00000001372 | ABCB11 | 80.96 | ENSOCUG00000021634 | TNFSF18 | 172.29 |

**Supplemental Table 2 Top 30 of downregulated genes after 3h vs control**

| noMMC |  |  | MMC |  |  |
| --- | --- | --- | --- | --- | --- |
| Gene ID | SYMBOL | Fold Change | Gene ID | SYMBOL | Fold Change |
| ENSOCUG00000010917 | SBSN | 0.0117 | ENSOCUG00000029646 | - | 0.0097 |
| ENSOCUG00000013038 | SLC24A5 | 0.0141 | ENSOCUG00000016111 | ANKEF1 | 0.0102 |
| ENSOCUG00000024509 | - | 0.0211 | ENSOCUG00000007796 | NDST4 | 0.0105 |
| ENSOCUG00000029361 | - | 0.0219 | ENSOCUG00000008583 | KLHL14 | 0.0113 |
| ENSOCUG00000000407 | DCT | 0.0307 | ENSOCUG00000034935 | - | 0.0157 |
| ENSOCUG00000038649 | - | 0.0338 | ENSOCUG00000003457 | CRYAA2 | 0.0164 |
| ENSOCUG00000029663 | KRT1 | 0.0393 | ENSOCUG00000030458 | - | 0.0172 |
| ENSOCUG00000028155 | SPATA3 | 0.0478 | ENSOCUG00000012663 | MSTN | 0.0176 |
| ENSOCUG00000030082 | C1orf100 | 0.0483 | ENSOCUG00000013576 | ENSA | 0.0188 |
| ENSOCUG00000029646 | - | 0.0496 | ENSOCUG00000029361 | - | 0.0195 |
| ENSOCUG00000009965 | TYR | 0.0593 | ENSOCUG00000033415 | - | 0.0204 |
| ENSOCUG00000006826 | IGFL4 | 0.0622 | ENSOCUG00000009754 | DNAAF11 | 0.0234 |
| ENSOCUG00000004221 | TYRP1 | 0.0635 | ENSOCUG00000002337 | PRR11 | 0.0256 |
| ENSOCUG00000034820 | - | 0.0662 | ENSOCUG00000003889 | PCARE | 0.0293 |
| ENSOCUG00000011173 | - | 0.0681 | ENSOCUG00000000031 | PBK | 0.0304 |
| ENSOCUG00000012952 | XCL1 | 0.0723 | ENSOCUG00000004582 | DMRT3 | 0.0305 |
| ENSOCUG00000007551 | TRPV6 | 0.0746 | ENSOCUG00000022067 | DLX4 | 0.0306 |
| ENSOCUG00000008197 | PCDH8 | 0.0748 | ENSOCUG00000038944 | C2orf16 | 0.0327 |
| ENSOCUG00000001296 | HES2 | 0.0751 | ENSOCUG00000026618 | - | 0.0356 |
| ENSOCUG00000011301 | FAM167A | 0.0753 | ENSOCUG00000031255 | - | 0.0357 |
| ENSOCUG00000000349 | TRIM46 | 0.0882 | ENSOCUG00000026559 | - | 0.0374 |
| ENSOCUG00000023409 | RAPGEFL1 | 0.0904 | ENSOCUG00000000349 | TRIM46 | 0.0379 |
| ENSOCUG00000023299 | RAB26 | 0.0910 | ENSOCUG00000026676 | - | 0.0380 |
| ENSOCUG00000026525 | CA8 | 0.0939 | ENSOCUG00000038682 | - | 0.0381 |
| ENSOCUG00000000670 | SLITRK6 | 0.0966 | ENSOCUG00000008197 | PCDH8 | 0.0383 |
| ENSOCUG00000016033 | WNT10A | 0.0984 | ENSOCUG00000038649 | - | 0.0390 |
| ENSOCUG00000001437 | S1PR5 | 0.0996 | ENSOCUG00000024181 | CCDC105 | 0.0390 |
| ENSOCUG00000032935 | - | 0.0997 | ENSOCUG00000016198 | MOGAT3 | 0.0413 |
| ENSOCUG00000014227 | SEC14L3 | 0.1047 | ENSOCUG00000001518 | LCA5L | 0.0431 |
| ENSOCUG00000011633 | MYB | 0.1061 | ENSOCUG00000007131 | FAM187B | 0.0454 |

**Supplemental Table 3 Top 30 of upregulated genes after 3d vs control**

| noMMC |  |  | MMC |  |  |
| --- | --- | --- | --- | --- | --- |
| Gene ID | SYMBOL | Fold Change | Gene ID | SYMBOL | Fold Change |
| ENSOCUG00000027187 | PRG4 | 2051.99 | ENSOCUG00000002481 | MMP13 | 20395.93 |
| ENSOCUG00000000666 | MAP3K19 | 826.20 | ENSOCUG00000029337 | MMP3 | 2665.34 |
| ENSOCUG00000022093 | ACAN | 785.77 | ENSOCUG00000013412 | CCL1 | 1487.23 |
| ENSOCUG00000000779 | RSPO2 | 696.97 | ENSOCUG00000027187 | PRG4 | 914.16 |
| ENSOCUG00000017620 | SAA2 | 611.25 | ENSOCUG00000004935 | CYP24A1 | 892.61 |
| ENSOCUG00000013367 | COL11A1 | 603.85 | ENSOCUG00000006901 | ANKRD1 | 730.79 |
| ENSOCUG00000011500 | TNC | 411.86 | ENSOCUG00000008771 | IL1A | 698.09 |
| ENSOCUG00000002481 | MMP13 | 331.70 | ENSOCUG00000034520 | ODAPH | 629.30 |
| ENSOCUG00000008086 | LRRC15 | 270.04 | ENSOCUG00000004126 | PLA2G2F | 534.82 |
| ENSOCUG00000011795 | SCN10A | 234.23 | ENSOCUG00000037423 | KRT6A | 450.37 |
| ENSOCUG00000015167 | CSN3 | 197.28 | ENSOCUG00000008086 | LRRC15 | 376.00 |
| ENSOCUG00000003792 | THBS1 | 184.76 | ENSOCUG00000011500 | TNC | 351.71 |
| ENSOCUG00000024881 | DIRAS3 | 179.49 | ENSOCUG00000024540 | - | 325.49 |
| ENSOCUG00000021257 | MATN3 | 176.10 | ENSOCUG00000000527 | DPYSL4 | 314.37 |
| ENSOCUG00000015320 | COL12A1 | 173.44 | ENSOCUG00000021580 | FGF23 | 308.16 |
| ENSOCUG00000026273 | SERPINA3 | 170.43 | ENSOCUG00000014282 | IL20 | 292.10 |
| ENSOCUG00000021580 | FGF23 | 157.52 | ENSOCUG00000005666 | DHRS9 | 245.66 |
| ENSOCUG00000008582 | HTR2B | 154.19 | ENSOCUG00000006660 | IL36G | 233.64 |
| ENSOCUG00000006901 | ANKRD1 | 125.60 | ENSOCUG00000016101 | MMP9 | 216.21 |
| ENSOCUG00000016068 | SERPINE1 | 122.07 | ENSOCUG00000026907 | MUC17 | 209.43 |
| ENSOCUG00000010061 | PLEKHG4 | 105.26 | ENSOCUG00000000779 | RSPO2 | 194.74 |
| ENSOCUG00000023263 | NDUFA4L2 | 95.92 | ENSOCUG00000006205 | OTOG | 194.01 |
| ENSOCUG00000022646 | S100A12 | 92.76 | ENSOCUG00000004530 | SERPINB13 | 146.43 |
| ENSOCUG00000016390 | CCN4 | 88.87 | ENSOCUG00000001813 | ARG1 | 142.39 |
| ENSOCUG00000006037 | ADGRG4 | 86.49 | ENSOCUG00000012042 | COCH | 138.90 |
| ENSOCUG00000003638 | GABRB3 | 85.11 | ENSOCUG00000034927 | CCL7 | 133.08 |
| ENSOCUG00000014502 | MC2R | 84.20 | ENSOCUG00000008031 | ETV4 | 123.38 |
| ENSOCUG00000012378 | SCG2 | 83.53 | ENSOCUG00000031236 | CPHXL | 122.26 |
| ENSOCUG00000026988 | CHI3L1 | 81.33 | ENSOCUG00000009679 | THEM5 | 121.35 |
| ENSOCUG00000008606 | FPR2 | 74.77 | ENSOCUG00000021476 | - | 118.61 |

**Supplemental Table 4 Top 30 of downregulated genes after 3d vs control**

| noMMC |  |  | MMC |  |  |
| --- | --- | --- | --- | --- | --- |
| Gene ID | SYMBOL | Fold Change | Gene ID | SYMBOL | Fold Change |
| ENSOCUG00000029574 | CYP2F1 | 0.0032 | ENSOCUG00000029574 | CYP2F1 | 0.0007 |
| ENSOCUG00000029646 | - | 0.0058 | ENSOCUG00000009583 | GRIK1 | 0.0023 |
| ENSOCUG00000006826 | IGFL4 | 0.0139 | ENSOCUG00000002467 | ZP4 | 0.0050 |
| ENSOCUG00000006337 | TRH | 0.0153 | ENSOCUG00000015957 | MYOC | 0.0067 |
| ENSOCUG00000002467 | ZP4 | 0.0161 | ENSOCUG00000006796 | CYP2A13 | 0.0068 |
| ENSOCUG00000035587 | - | 0.0170 | ENSOCUG00000014227 | SEC14L3 | 0.0068 |
| ENSOCUG00000029429 | SULT1E1 | 0.0196 | ENSOCUG00000029646 | - | 0.0074 |
| ENSOCUG00000007567 | SLC38A11 | 0.0199 | ENSOCUG00000013265 | PLA2G2A | 0.0078 |
| ENSOCUG00000008589 | C1orf194 | 0.0199 | ENSOCUG00000011172 | OPCML | 0.0080 |
| ENSOCUG00000010917 | SBSN | 0.0205 | ENSOCUG00000002462 | SLC24A2 | 0.0087 |
| ENSOCUG00000011548 | MYOG | 0.0209 | ENSOCUG00000016780 | GRIA1 | 0.0090 |
| ENSOCUG00000001988 | SPRR3 | 0.0224 | ENSOCUG00000005321 | SYT13 | 0.0100 |
| ENSOCUG00000009920 | TMPRSS7 | 0.0230 | ENSOCUG00000001473 | S100B | 0.0124 |
| ENSOCUG00000006362 | SLC25A48 | 0.0243 | ENSOCUG00000013536 | NTRK3 | 0.0137 |
| ENSOCUG00000000761 | NELL1 | 0.0267 | ENSOCUG00000009269 | NLGN1 | 0.0142 |
| ENSOCUG00000025191 | - | 0.0278 | ENSOCUG00000004245 | CLCNKA | 0.0146 |
| ENSOCUG00000016629 | SSC4D | 0.0280 | ENSOCUG00000009464 | ADGRB3 | 0.0148 |
| ENSOCUG00000005933 | ACSM2A | 0.0284 | ENSOCUG00000012056 | CHODL | 0.0149 |
| ENSOCUG00000013555 | PPP1R1A | 0.0295 | ENSOCUG00000013555 | PPP1R1A | 0.0165 |
| ENSOCUG00000010184 | TREH | 0.0303 | ENSOCUG00000010184 | TREH | 0.0167 |
| ENSOCUG00000008917 | C6orf58 | 0.0326 | ENSOCUG00000006361 | FAM180B | 0.0170 |
| ENSOCUG00000022472 | - | 0.0384 | ENSOCUG00000000072 | CBLN4 | 0.0189 |
| ENSOCUG00000005177 | FMO2 | 0.0429 | ENSOCUG00000014890 | PAH | 0.0192 |
| ENSOCUG00000029014 | C1orf105 | 0.0433 | ENSOCUG00000009790 | ERBB4 | 0.0193 |
| ENSOCUG00000010912 | KRTDAP | 0.0462 | ENSOCUG00000027090 | IGLV5-52 | 0.0199 |
| ENSOCUG00000001201 | EDN2 | 0.0464 | ENSOCUG00000005177 | FMO2 | 0.0203 |
| ENSOCUG00000027883 | KIAA0408 | 0.0486 | ENSOCUG00000006124 | LNP1 | 0.0203 |
| ENSOCUG00000017861 | LRAT | 0.0505 | ENSOCUG00000016289 | HPGD | 0.0207 |
| ENSOCUG00000012252 | DPP6 | 0.0515 | ENSOCUG00000003889 | PCARE | 0.0223 |
| ENSOCUG00000007470 | ZBTB16 | 0.0520 | ENSOCUG00000002203 | PTN | 0.0232 |

**Supplemental Table 5 Top 30 of upregulated genes after 14d vs control**

| noMMC |  |  | MMC |  |  |
| --- | --- | --- | --- | --- | --- |
| Gene ID | SYMBOL | Fold Change | Gene ID | SYMBOL | Fold Change |
| ENSOCUG00000013576 | ENSA | 668.47 | ENSOCUG00000008086 | LRRC15 | 2030.54 |
| ENSOCUG00000012720 | TRIM58 | 232.81 | ENSOCUG00000008303 | MMP12 | 1787.93 |
| ENSOCUG00000002481 | MMP13 | 159.17 | ENSOCUG00000016101 | MMP9 | 838.15 |
| ENSOCUG00000002435 | HBQ1 | 151.24 | ENSOCUG00000011500 | TNC | 472.72 |
| ENSOCUG00000010701 | HEMGN | 149.90 | ENSOCUG00000003353 | CNGA3 | 440.34 |
| ENSOCUG00000000568 | - | 149.00 | ENSOCUG00000008858 | CORIN | 291.61 |
| ENSOCUG00000013603 | ALAS2 | 148.15 | ENSOCUG00000013367 | COL11A1 | 271.96 |
| ENSOCUG00000022340 | - | 146.00 | ENSOCUG00000029337 | MMP3 | 262.72 |
| ENSOCUG00000011988 | CYP2D6 | 114.09 | ENSOCUG00000021307 | DEFA3 | 242.58 |
| ENSOCUG00000031685 | - | 110.76 | ENSOCUG00000011051 | TNFSF11 | 138.56 |
| ENSOCUG00000014162 | HBZ | 107.97 | ENSOCUG00000036803 | TMEM132B | 131.68 |
| ENSOCUG00000033540 | - | 101.70 | ENSOCUG00000034082 | CR2 | 130.39 |
| ENSOCUG00000029727 | TUBB1 | 100.93 | ENSOCUG00000022093 | ACAN | 125.19 |
| ENSOCUG00000000779 | RSPO2 | 94.34 | ENSOCUG00000017064 | UPK3A | 122.34 |
| ENSOCUG00000005039 | CA1 | 90.15 | ENSOCUG00000005746 | KLK4 | 112.14 |
| ENSOCUG00000023449 | HMGB2 | 87.22 | ENSOCUG00000000666 | MAP3K19 | 94.46 |
| ENSOCUG00000017459 | EPB42 | 73.01 | ENSOCUG00000009672 | PLET1 | 85.03 |
| ENSOCUG00000013367 | COL11A1 | 69.74 | ENSOCUG00000025675 | EDN3 | 76.54 |
| ENSOCUG00000008303 | MMP12 | 65.10 | ENSOCUG00000021849 | - | 76.08 |
| ENSOCUG00000006884 | SLFN14 | 58.73 | ENSOCUG00000009023 | FCRL4 | 75.89 |
| ENSOCUG00000026988 | CHI3L1 | 47.91 | ENSOCUG00000008031 | ETV4 | 69.93 |
| ENSOCUG00000001856 | CTSE | 42.97 | ENSOCUG00000029569 | KRT17 | 60.88 |
| ENSOCUG00000007440 | ITGAD | 39.43 | ENSOCUG00000013688 | TNFAIP6 | 56.61 |
| ENSOCUG00000009244 | THBS4 | 37.40 | ENSOCUG00000016337 | CD300LF | 51.25 |
| ENSOCUG00000004499 | SLC4A1 | 37.00 | ENSOCUG00000024540 | - | 45.50 |
| ENSOCUG00000017905 | PCK1 | 36.68 | ENSOCUG00000009487 | TRPC3 | 45.22 |
| ENSOCUG00000016101 | MMP9 | 34.07 | ENSOCUG00000016390 | CCN4 | 44.53 |
| ENSOCUG00000013389 | EPYC | 33.00 | ENSOCUG00000026206 | - | 43.03 |
| ENSOCUG00000027187 | PRG4 | 32.60 | ENSOCUG00000017338 | EDIL3 | 42.53 |
| ENSOCUG00000009582 | CLEC5A | 30.76 | ENSOCUG00000011970 | TAGLN | 39.06 |

**Supplemental Table 6 Top 30 of downregulated genes after 14d vs control**

| noMMC |  |  | MMC |  |  |
| --- | --- | --- | --- | --- | --- |
| Gene ID | SYMBOL | Fold Change | Gene ID | SYMBOL | Fold Change |
| ENSOCUG00000013038 | SLC24A5 | 0.0063 | ENSOCUG00000005933 | ACSM2A | 0.0010 |
| ENSOCUG00000001988 | SPRR3 | 0.0087 | ENSOCUG00000026487 | - | 0.0098 |
| ENSOCUG00000010917 | SBSN | 0.0103 | ENSOCUG00000011172 | OPCML | 0.0176 |
| ENSOCUG00000010912 | KRTDAP | 0.0359 | ENSOCUG00000009056 | PDILT | 0.0182 |
| ENSOCUG00000025593 | IVL | 0.0445 | ENSOCUG00000002173 | PYGM | 0.0535 |
| ENSOCUG00000003510 | OCA2 | 0.0508 | ENSOCUG00000023508 | - | 0.0899 |
| ENSOCUG00000007282 | SVOPL | 0.0845 | ENSOCUG00000009058 | HPR | 0.1741 |
| ENSOCUG00000000407 | DCT | 0.0851 | ENSOCUG00000028024 | IRGM | 0.2429 |
| ENSOCUG00000029663 | KRT1 | 0.1072 | ENSOCUG00000016911 | TDRD12 | 0.2715 |
| ENSOCUG00000022951 | - | 0.1166 | ENSOCUG00000014904 | EPAS1 | 0.2907 |
| ENSOCUG00000016374 | MAK | 0.1294 | ENSOCUG00000017309 | HTR4 | 0.2955 |
| ENSOCUG00000030272 | - | 0.1313 | ENSOCUG00000013831 | TMOD1 | 0.3343 |
| ENSOCUG00000000999 | PMEL | 0.1351 | ENSOCUG00000029619 | ZNF302 | 0.4501 |
| ENSOCUG00000024935 | PDZK1 | 0.1492 |  |  |  |
| ENSOCUG00000008748 | NT5DC4 | 0.1545 |  |  |  |
| ENSOCUG00000014227 | SEC14L3 | 0.1546 |  |  |  |
| ENSOCUG00000028024 | IRGM | 0.1625 |  |  |  |
| ENSOCUG00000034737 | - | 0.1982 |  |  |  |
| ENSOCUG00000029183 | UBAP1L | 0.2008 |  |  |  |
| ENSOCUG00000012065 | PTPRR | 0.2031 |  |  |  |
| ENSOCUG00000008779 | SPINK5 | 0.2144 |  |  |  |
| ENSOCUG00000039130 | - | 0.2203 |  |  |  |
| ENSOCUG00000017861 | LRAT | 0.2324 |  |  |  |
| ENSOCUG00000021090 | - | 0.2338 |  |  |  |
| ENSOCUG00000014012 | A2ML1 | 0.2402 |  |  |  |
| ENSOCUG00000036714 | MPIG6B | 0.2536 |  |  |  |
| ENSOCUG00000033457 | - | 0.2584 |  |  |  |
| ENSOCUG00000004655 | CYP2B6 | 0.2691 |  |  |  |
| ENSOCUG00000014728 | MUC16 | 0.2733 |  |  |  |
| ENSOCUG00000003486 | SLC5A8 | 0.2970 |  |  |  |

**Supplemental Table 7 Top 30 of significantly changed genes at 3h after GFS - Comparison with GFS without MMC**

| up |  |  | down |  |  |
| --- | --- | --- | --- | --- | --- |
| Gene ID | SYMBOL | Fold Change | Gene ID | SYMBOL | Fold Change |
| ENSOCUG00000034917 | RN7SL591P | 59.83 | ENSOCUG00000026636 | MYH1 | 0.0000010 |
| ENSOCUG00000008606 | FPR2 | 54.90 | ENSOCUG00000010207 | MYH13 | 0.0000017 |
| ENSOCUG00000012764 | KRT76 | 53.40 | ENSOCUG00000029652 | MYH4 | 0.0000030 |
| ENSOCUG00000003344 | IDO1 | 49.10 | ENSOCUG00000014182 | MYL1 | 0.0000033 |
| ENSOCUG00000033407 | - | 46.08 | ENSOCUG00000004509 | - | 0.0000056 |
| ENSOCUG00000025638 | CXCR2 | 45.91 | ENSOCUG00000004074 | ACTC1 | 0.0000058 |
| ENSOCUG00000030497 | - | 36.80 | ENSOCUG00000025586 | MYL2 | 0.0000152 |
| ENSOCUG00000021580 | FGF23 | 34.31 | ENSOCUG00000027154 | CKM | 0.0000180 |
| ENSOCUG00000012495 | LIPN | 32.71 | ENSOCUG00000014414 | MYOT | 0.0000247 |
| ENSOCUG00000038024 | GIMAP4 | 30.03 | ENSOCUG00000025160 | TCAP | 0.0000282 |
| ENSOCUG00000006280 | CDSN | 29.69 | ENSOCUG00000008064 | TRDN | 0.0000311 |
| ENSOCUG00000007303 | CHRNA10 | 29.45 | ENSOCUG00000011037 | MYH7 | 0.0000413 |
| ENSOCUG00000025716 | - | 28.73 | ENSOCUG00000010229 | MYH8 | 0.0000442 |
| ENSOCUG00000011554 | - | 26.73 | ENSOCUG00000017551 | XIRP2 | 0.0000460 |
| ENSOCUG00000000797 | VSTM1 | 25.69 | ENSOCUG00000008959 | MYOZ2 | 0.0000550 |
| ENSOCUG00000008177 | MGAM | 25.25 | ENSOCUG00000025140 | SRL | 0.0000562 |
| ENSOCUG00000009670 | NLRP12 | 24.83 | ENSOCUG00000012560 | MYOZ1 | 0.0000598 |
| ENSOCUG00000004022 | - | 23.05 | ENSOCUG00000004480 | CSRP3 | 0.0000620 |
| ENSOCUG00000017210 | NPTX2 | 20.56 | ENSOCUG00000011023 | NRAP | 0.0000674 |
| ENSOCUG00000009195 | CLEC4E | 20.37 | ENSOCUG00000026126 | MB | 0.0000709 |
| ENSOCUG00000015108 | CXCR1 | 20.34 | ENSOCUG00000003619 | CACNA1S | 0.0000809 |
| ENSOCUG00000012798 | - | 20.13 | ENSOCUG00000006159 | MYH15 | 0.0000809 |
| ENSOCUG00000027783 | CCNI | 19.98 | ENSOCUG00000001849 | SMPX | 0.0000822 |
| ENSOCUG00000001327 | HRH4 | 19.37 | ENSOCUG00000027208 | MYBPHL | 0.0000841 |
| ENSOCUG00000026468 | BDKRB1 | 18.84 | ENSOCUG00000012392 | MYL3 | 0.0000886 |
| ENSOCUG00000030664 | - | 18.20 | ENSOCUG00000006570 | ABRA | 0.0000959 |
| ENSOCUG00000007268 | TREML2 | 17.41 | ENSOCUG00000007837 | LRRC39 | 0.0000988 |
| ENSOCUG00000015833 | SAMSN1 | 17.33 | ENSOCUG00000000303 | PPP1R3A | 0.0000997 |
| ENSOCUG00000014907 | WIF1 | 16.70 | ENSOCUG00000029864 | STRIT1 | 0.0001226 |
| ENSOCUG00000023676 | MSMB | 16.52 | ENSOCUG00000006209 | HJV | 0.0001323 |

**Supplemental Table 8 Top 30 of significantly changed gene at 3d after GFS - Comparison with GFS without MMC**

| up |  |  | down |  |  |
| --- | --- | --- | --- | --- | --- |
| Gene ID | Symbol | Fold Change | Gene ID | Symbol | Fold Change |
| ENSOCUG00000004126 | PLA2G2F | 251.70 | ENSOCUG00000015957 | MYOC | 0.0036984 |
| ENSOCUG00000004935 | CYP24A1 | 154.51 | ENSOCUG00000001473 | S100B | 0.0081569 |
| ENSOCUG00000005933 | ACSM2A | 144.59 | ENSOCUG00000024881 | DIRAS3 | 0.0127092 |
| ENSOCUG00000007393 | - | 88.44 | ENSOCUG00000002089 | PRND | 0.0138312 |
| ENSOCUG00000005666 | DHRS9 | 82.94 | ENSOCUG00000029199 | DHRS3 | 0.0159312 |
| ENSOCUG00000035413 | - | 78.22 | ENSOCUG00000006989 | RAG1 | 0.0163332 |
| ENSOCUG00000029337 | MMP3 | 74.80 | ENSOCUG00000027689 | ASIC3 | 0.0168203 |
| ENSOCUG00000014805 | WNT3 | 68.77 | ENSOCUG00000007382 | INHBE | 0.0208315 |
| ENSOCUG00000011744 | IGSF9B | 67.46 | ENSOCUG00000004247 | SGCZ | 0.0216923 |
| ENSOCUG00000015695 | AVPR2 | 57.77 | ENSOCUG00000012959 | ADAMTS20 | 0.0219788 |
| ENSOCUG00000009679 | THEM5 | 50.70 | ENSOCUG00000006029 | A2M | 0.0239605 |
| ENSOCUG00000026870 | - | 46.73 | ENSOCUG00000017002 | MDGA2 | 0.0240143 |
| ENSOCUG00000007555 | PGLYRP2 | 45.22 | ENSOCUG00000036058 | - | 0.0241135 |
| ENSOCUG00000038395 | - | 45.07 | ENSOCUG00000009269 | NLGN1 | 0.0266046 |
| ENSOCUG00000008126 | LDLRAD1 | 44.86 | ENSOCUG00000035779 | - | 0.0269136 |
| ENSOCUG00000024590 | MAGEA10 | 44.71 | ENSOCUG00000002462 | SLC24A2 | 0.0271645 |
| ENSOCUG00000021234 | - | 43.91 | ENSOCUG00000015167 | CSN3 | 0.0281372 |
| ENSOCUG00000004216 | SLC6A20 | 42.04 | ENSOCUG00000033477 | - | 0.0288775 |
| ENSOCUG00000034532 | - | 41.91 | ENSOCUG00000025689 | RTL3 | 0.0292892 |
| ENSOCUG00000012042 | COCH | 41.41 | ENSOCUG00000008486 | CLVS2 | 0.0294741 |
| ENSOCUG00000009384 | CD8B2 | 41.23 | ENSOCUG00000017620 | SAA1 | 0.0326228 |
| ENSOCUG00000004530 | SERPINB13 | 38.98 | ENSOCUG00000000666 | MAP3K19 | 0.0335009 |
| ENSOCUG00000031236 | CPHXL | 38.87 | ENSOCUG00000037248 | GPC6 | 0.0354049 |
| ENSOCUG00000029792 | G3BP1 | 38.20 | ENSOCUG00000006752 | MEST | 0.0382159 |
| ENSOCUG00000025675 | EDN3 | 36.66 | ENSOCUG00000016920 | NSUN7 | 0.0386581 |
| ENSOCUG00000000527 | DPYSL4 | 35.98 | ENSOCUG00000005743 | - | 0.0414879 |
| ENSOCUG00000035319 | - | 34.73 | ENSOCUG00000013451 | RLN3 | 0.0416573 |
| ENSOCUG00000014781 | - | 33.54 | ENSOCUG00000028035 | ASB18 | 0.0417422 |
| ENSOCUG00000027498 | BTG4 | 30.30 | ENSOCUG00000035651 | - | 0.0418764 |
| ENSOCUG00000011451 | - | 30.10 | ENSOCUG00000037069 | - | 0.0422133 |

**Supplemental Table 9 Top 30 of significantly changed genes at 14d after GFS - Comparison with GFS without MMC**

| up |  |  | down |  |  |
| --- | --- | --- | --- | --- | --- |
| Gene ID | SYMBOL | Fold Change | Gene ID | SYMBOL | Fold Change |
| ENSOCUG00000000330 | R3HDML | 1494.08 | ENSOCUG00000026636 | MYH1 | 0.0000026 |
| ENSOCUG00000005746 | KLK4 | 580.10 | ENSOCUG00000029652 | MYH4 | 0.0000042 |
| ENSOCUG00000003353 | CNGA3 | 435.43 | ENSOCUG00000014182 | MYL1 | 0.0000103 |
| ENSOCUG00000027218 | - | 270.15 | ENSOCUG00000017551 | XIRP2 | 0.0000209 |
| ENSOCUG00000005743 | - | 91.13 | ENSOCUG00000025586 | MYL2 | 0.0000230 |
| ENSOCUG00000025408 | ULBP3 | 90.66 | ENSOCUG00000012560 | MYOZ1 | 0.0000295 |
| ENSOCUG00000003433 | CSF2 | 88.02 | ENSOCUG00000008064 | TRDN | 0.0000342 |
| ENSOCUG00000004842 | BHMT2 | 62.90 | ENSOCUG00000017131 | LMOD2 | 0.0000452 |
| ENSOCUG00000004324 | ZFP42 | 60.83 | ENSOCUG00000026126 | MB | 0.0000496 |
| ENSOCUG00000034520 | ODAPH | 59.43 | ENSOCUG00000025160 | TCAP | 0.0000514 |
| ENSOCUG00000032617 | - | 57.71 | ENSOCUG00000010229 | MYH8 | 0.0000654 |
| ENSOCUG00000023958 | IL1B | 57.04 | ENSOCUG00000023462 | MYLPF | 0.0000665 |
| ENSOCUG00000006660 | IL36G | 56.27 | ENSOCUG00000027154 | CKM | 0.0000699 |
| ENSOCUG00000011051 | TNFSF11 | 55.71 | ENSOCUG00000000303 | PPP1R3A | 0.0000809 |
| ENSOCUG00000029569 | KRT17 | 51.53 | ENSOCUG00000010207 | MYH13 | 0.0000829 |
| ENSOCUG00000026787 | DCDC2C | 47.52 | ENSOCUG00000008959 | MYOZ2 | 0.0000874 |
| ENSOCUG00000021307 | DEFA6 | 46.12 | ENSOCUG00000011037 | MYH7 | 0.0001097 |
| ENSOCUG00000014572 | DEFA1 | 44.84 | ENSOCUG00000014414 | MYOT | 0.0001107 |
| ENSOCUG00000003616 | CD70 | 44.60 | ENSOCUG00000001849 | SMPX | 0.0001119 |
| ENSOCUG00000002612 | KRT84 | 43.44 | ENSOCUG00000004074 | ACTC1 | 0.0001214 |
| ENSOCUG00000026206 | - | 40.15 | ENSOCUG00000027208 | MYBPHL | 0.0001303 |
| ENSOCUG00000000239 | ATP6V1G3 | 38.73 | ENSOCUG00000030778 | - | 0.0001319 |
| ENSOCUG00000030078 | - | 37.28 | ENSOCUG00000026544 | - | 0.0001319 |
| ENSOCUG00000011835 | CXCL8 | 35.81 | ENSOCUG00000002688 | ATP2A1 | 0.0001320 |
| ENSOCUG00000011843 | HSD11B2 | 33.79 | ENSOCUG00000016949 | SMYD1 | 0.0001358 |
| ENSOCUG00000011143 | FOSL1 | 32.83 | ENSOCUG00000007837 | LRRC39 | 0.0001429 |
| ENSOCUG00000009384 | CD8B2 | 31.70 | ENSOCUG00000007566 | ACTA1 | 0.0001486 |
| ENSOCUG00000025297 | H2AC11 | 30.87 | ENSOCUG00000011023 | NRAP | 0.0001531 |
| ENSOCUG00000016101 | MMP9 | 30.55 | ENSOCUG00000012392 | MYL3 | 0.0001763 |
| ENSOCUG00000017739 | BRINP2 | 30.40 | ENSOCUG00000016777 | - | 0.0001784 |

**Supplemental Table 10 Enrichment analysis using MSigDB Hallmark at 3h-up**

| Group | Gene_set_name | ID | Count | GeneRatio | qvalue | GeneSymbol |
| --- | --- | --- | --- | --- | --- | --- |
| Up | Epithelial mesenchymal transition | M5930 | 71 | 0.17150 | 7.05E-24 | WNT5A/COL6A2/INHBA/FLNA/PDGFRB/EMP3/MFAP5/VCAN/IGFBP4/CCN2/SERPINH1/LOX/THBS1/CCN1/GLIPR1/FSTL1/THBS2/ITGB3/LUM/GREM1/COL16A1/FBLN2/MMP2/DCN/GPX7/THY1/LRRC15/ECM2/TIMP3/IGFBP2/GADD45A/VIM/GJA1/FN1/PTX3/TIMP1/FBLN1/ITGA2/POSTN/DKK1/CXCL8/SPARC/COL1A2/SCG2/COL1A1/PCOLCE2/FBLN5/HTRA1/PTHLH/COL3A1/COL5A2/SERPINE1/TGFBR3/MGP/SLIT3/MATN2/MMP1/PLOD3/PDLIM4/BGN/COL5A1/SDC1/PVR/PLAUR/TNFRSF12A/FZD8/PMEPA1/PCOLCE/ELN/RHOB/COL6A3 |
| Up | Inflammatory response | M5932 | 54 | 0.13043 | 2.61E-12 | PCDH7/LCP2/INHBA/GPC3/EMP3/IRF7/KLF6/IRAK2/LIF/CD14/BTG2/NLRP3/ITGB3/PDPN/SPHK1/FPR1/IL1A/CLEC5A/AQP9/ADM/OSM/TIMP1/IL7R/CXCL8/CHST2/NOD2/SLC7A1/CD48/TNFAIP6/TNFRSF1B/MSR1/MXD1/CDKN1A/SERPINE1/SELL/RHOG/HIF1A/PTAFR/CCL2/CCRL2/C5AR1/PIK3R5/SLAMF1/IL1B/CSF1/PVR/BDKRB1/PTGIR/HAS2/PLAUR/FZD5/ICAM1/CCL7/PTPRE |
| Up | TNFa signaling via NFkB | M5890 | 49 | 0.11836 | 1.22E-09 | DUSP2/NFIL3/EGR3/INHBA/SLC16A6/ZBTB10/KLF6/ZFP36/DUSP4/LIF/CCN1/LAMB3/NFAT5/BTG2/YRDC/PLEK/MAFF/SPHK1/TNF/IL1A/GADD45A/TUBB2A/PTX3/FOSL2/ACKR3/FOSL1/IL7R/EGR2/TNFAIP6/BTG3/MXD1/CDKN1A/SERPINE1/IER3/DRAM1/PTGS2/FOS/CCL2/CCRL2/PHLDA1/IL1B/TRIB1/CSF1/FJX1/PLAUR/ICAM1/PMEPA1/RHOB/PTPRE |
| Up | Apoptosis | M5902 | 35 | 0.08454 | 1.12E-05 | EGR3/IGFBP6/HGF/PDGFRB/SLC20A1/CD14/BTG2/TSPO/LUM/TNF/MMP2/PRF1/DCN/TIMP3/IL1A/GADD45A/PLAT/HMOX1/TIMP1/KRT18/BTG3/NEDD9/ANXA1/CASP9/CDKN1A/TGFBR3/IER3/CD38/F2R/BGN/IL1B/SMAD7/TNFRSF12A/RHOB/H1-0 |
| Up | Coagulation | M5946 | 31 | 0.07488 | 1.77E-05 | CTSE/DUSP6/THBS1/PRSS23/ITGB3/PLEK/CTSH/MAFF/MMP2/TIMP3/KLF7/PLAT/FN1/TIMP1/CTSK/ITGA2/SPARC/HTRA1/CPQ/C1S/C1R/PECAM1/ANXA1/CASP9/SERPINE1/MMP9/MMP1/FURIN/PROS1/SERPING1/CFB |
| Up | Hypoxia | M5891 | 39 | 0.09420 | 2.77E-05 | NFIL3/GPC3/KLF6/CCN2/LOX/ZFP36/CCN1/ERRFI1/STC1/MAFF/DCN/CAVIN3/SRPX/NAGK/P4HA2/HEXA/KLF7/ADM/HMOX1/FOSL2/ACKR3/PFKP/HSPA5/IDS/PIM1/CHST2/PLIN2/CDKN1A/SERPINE1/TMEM45A/IER3/ANGPTL4/FOS/CHST3/CITED2/BGN/COL5A1/CCN5/PLAUR |
| Up | KRas signaling up | M5953 | 39 | 0.09420 | 2.77E-05 | INHBA/ARG1/DUSP6/LIF/YRDC/KCNN4/TFPI/ITGBL1/RELN/FBXO4/PLAT/RBP4/CLEC4A/FLT4/SPON1/ITGA2/IL7R/ITGB2/TLR8/ADGRA2/WNT7A/TNFRSF1B/IKZF1/PECAM1/MMP9/ANGPTL4/PTGS2/LCP1/MAFB/CSF2RA/APOD/IL1B/TRIB1/IL2RG/SERPINA3/GPNMB/PLAUR/CD37/CFB |
| Up | IL6 JAK STAT3 signaling | M5897 | 21 | 0.05072 | 1.50E-04 | CNTFR/MYD88/ITGA4/CCR1/CD14/ITGB3/TNF/HMOX1/PIM1/PLA2G2A/TNFRSF1B/IL1R2/CD38/PIK3R5/CSF2RA/IL1B/CSF1/IL2RG/CSF2RB/TNFRSF12A/CCL7 |
| Up | p53 pathway | M5939 | 35 | 0.08454 | 6.77E-04 | TRAF4/SLC3A2/SLC7A11/TOB1/LIF/BTG2/ZMAT3/SPHK1/IL1A/GADD45A/ADA/HMOX1/XPC/CYFIP2/PLK3/RAP2B/DEF6/MXD1/GM2A/IRAG2/CDKN1A/PRKAB1/IER3/DRAM1/PROCR/FOS/F2R/CEBPA/RPL18/TAX1BP3/SLC19A2/SDC1/AEN/KRT17/PTPRE |
| Up | Allograft rejection | M5950 | 33 | 0.07971 | 2.78E-03 | LCP2/INHBA/FLNA/IRF7/PTPN6/CCR1/LIF/IGSF6/NLRP3/TNF/PRF1/THY1/GPR65/FGR/TIMP1/HLA-E/ITGB2/PTPRC/HCLS1/FYB1/ITGAL/GZMA/CFP/MMP9/HIF1A/CCL2/F2R/IL1B/SRGN/CSF1/IL2RG/ICAM1/CCL7 |
| Up | Apical junction | M5915 | 31 | 0.07488 | 9.95E-03 | NECTIN3/VCAN/LAMB3/CLDN11/COL16A1/ACTB/CDSN/MMP2/IRS1/THY1/CNTN1/THBS3/SKAP2/ITGA2/PTPRC/FYB1/PKD1/INSIG1/PECAM1/MMP9/RAC2/CADM3/SYK/NECTIN2/CD276/SIRPA/ZYX/FSCN1/GRB7/GTF2F1/ICAM1 |
| Up | IL2 STAT5 signaling | M5947 | 30 | 0.07246 | 1.47E-02 | NFIL3/CKAP4/UMPS/ADAM19/PRNP/KLF6/SPRY4/LIF/ODC1/RHOH/MAFF/PENK/IKZF2/GPR65/SLC1A5/LRRC8C/PIM1/PLIN2/CD48/FGL2/TNFRSF1B/MXD1/IL1R2/SELL/PHLDA1/FURIN/CSF1/AHNAK/RHOB/PRAF2 |
| Up | Complement | M5921 | 30 | 0.07246 | 1.47E-02 | LCP2/ITGAM/IRF7/MMP13/SH2B3/DUSP6/CASP10/PLEK/CTSH/MAFF/PLAT/FN1/TIMP1/HSPA5/PIM1/CPQ/C1S/C1R/GZMA/CASP9/SERPINE1/RHOG/PIK3R5/FCN1/SERPING1/F5/PLAUR/CFB/HSPA1A/ATOX1 |
| Up | mTORC1 signaling | M5924 | 30 | 0.07246 | 1.47E-02 | NFIL3/SLC7A11/CORO1A/SERPINH1/HMGCS1/BTG2/CYP51A1/MTHFD2/STC1/PSAT1/TMEM97/G6PD/SLC1A5/RRP9/SLC1A4/SKAP2/ITGB2/HSPA5/FGL2/SYTL2/INSIG1/EDEM1/NIBAN1/IFRD1/SQLE/CFP/CDKN1A/HMGCR/SLA/NFKBIB |
| Up | Cholesterol homeostasis | M5892 | 14 | 0.03382 | 1.75E-02 | NFIL3/HMGCS1/CYP51A1/ERRFI1/ANTXR2/TMEM97/ACAT2/NIBAN1/GPX8/SQLE/TP53INP1/HMGCR/PLAUR/TNFRSF12A |
| Up | TGF beta signaling | M5896 | 11 | 0.02657 | 2.10E-02 | SLC20A1/THBS1/SKIL/LTBP2/SERPINE1/TGFBR1/ID3/SMAD7/ID1/FURIN/PMEPA1 |
| Up | Interferon gamma response | M5913 | 29 | 0.07005 | 2.19E-02 | LCP2/ITGB7/IRF7/MYD88/IDO1/PTPN6/MX1/MTHFD2/FPR1/PFKP/MARCHF1/PIM1/RSAD2/FGL2/TNFAIP6/C1S/C1R/GZMA/CDKN1A/PTGS2/HIF1A/CD38/CCL2/SERPING1/CSF2RB/ISG15/ICAM1/CFB/CCL7 |
| Up | Angiogenesis | M5944 | 8 | 0.01932 | 2.95E-02 | VCAN/FSTL1/LUM/STC1/TIMP1/POSTN/COL3A1/COL5A2 |

**Supplemental Table 11 Enrichment analysis using MSigDB Hallmark at 3h-down**

| Group | Gene_set_name | ID | Count | GeneRatio | qvalue | GeneSymbol |
| --- | --- | --- | --- | --- | --- | --- |
| Down | Myogenesis | M5909 | 85 | 0.19495 | 5.36E-34 | DES/SCD/MYF6/LARGE1/TNNT3/SGCD/ENO3/PYGM/CD36/ATP2A1/LPIN1/DTNA/SGCG/ACTC1/CSRP3/HSPB8/AK1/CAV3/NCAM1/CHRNB1/COX6A2/MAPK12/ACTA1/ANKRD2/ADCY9/TNNC1/MYOM1/CACNG1/AGL/CASQ1/REEP1/TPM2/MYH8/TNNI1/CASQ2/SSPN/MYL4/SVIL/MYBPH/MYH7/CKMT2/PDE4DIP/SCHIP1/MYOG/TPD52L1/MYL3/MYOZ1/LDB3/COX7A1/VIPR1/MYOM2/MYH11/SORBS1/MYL1/SPEG/FABP3/MAPRE3/BIN1/MYLK/PPP1R3C/TNNT2/PFKM/ACTN2/PKIA/ITGA7/DMD/RYR1/ACTN3/MYL11/CHRNG/CHRNA1/TCAP/PTP4A3/MYL2/MB/MYH1/SGCA/CKM/PVALB/KCNH2/PPFIA4/PGAM2/MEF2C/MYH4/CRAT |
| Down | Oxidative phosphorylation | M5936 | 71 | 0.16284 | 1.37E-22 | DLD/ECHS1/ETFA/NDUFS4/NDUFB8/ATP5F1E/SLC25A11/NNT/GPI/COX4I1/ACAT1/AIFM1/COX15/COX5A/SUCLG1/GOT2/UQCRC2/ATP5PO/NDUFC1/NDUFS6/ATP5F1C/ACADSB/MDH1/DECR1/NDUFS3/ETFDH/ATP5PF/ATP1B1/CS/PDHX/UQCRB/NDUFAB1/NDUFA7/NDUFV2/IMMT/IDH2/NDUFB3/SLC25A12/NDUFS1/RHOT1/NDUFA8/NDUFS2/UQCRQ/ACADM/MDH2/NDUFA9/PHYH/NDUFA6/DLAT/PDHB/ATP5MF/ATP5MC1/SLC25A20/COX5B/PDK4/NDUFB5/ACO2/NDUFA3/MPC1/SDHA/ATP5ME/SLC25A4/ALDH6A1/UQCRFS1/ATP5MG/NDUFA5/SDHC/SDHB/COX7B/NDUFA1/COX6C |
| Down | Adipogenesis | M5905 | 49 | 0.11239 | 1.13E-08 | DLD/ECHS1/GPHN/DHRS7/ELMOD3/CD36/AIFM1/ANGPT1/SUCLG1/ATP5PO/HSPB8/HIBCH/GBE1/CMBL/SPARCL1/ALDOA/DECR1/NDUFS3/CS/CDKN2C/NDUFAB1/IMMT/LEP/SSPN/ME1/MCCC1/PPARG/SNCG/UQCRQ/SORBS1/ACADM/HADH/MDH2/COQ5/MYLK/COQ9/PHYH/DLAT/CPT2/ACO2/ITGA7/ADIPOQ/NDUFA5/CRAT/SDHC/COQ3/SDHB/COX7B/PGM1 |
| Down | Fatty acid metabolism | M5935 | 41 | 0.09404 | 3.73E-08 | DLD/ECHS1/ENO3/CD36/CRYZ/SUCLG1/HIBCH/HMGCS2/MDH1/ALDOA/DECR1/ETFDH/AUH/MCEE/PPARA/CA4/BPHL/ME1/AADAT/CA2/GCDH/EHHADH/SUCLG2/ACSS1/BCKDHB/ACADM/HADH/MDH2/BMPR1B/PDHB/CPT2/HSDL2/CYP1A1/CBR1/ACO2/D2HGDH/SDHA/GSTZ1/CRAT/SDHC/AOC3 |

**Supplemental Table 12 Enrichment analysis using MSigDB Hallmark at 3d**

| Group | Gene_set_name | ID | Count | GeneRatio | qvalue | GeneSymbol |
| --- | --- | --- | --- | --- | --- | --- |
| Up | Interferon gamma response | M5913 | 73 | 0.16553 | 1.41E-23 | LCP2/IRF8/NLRC5/SAMD9L/EPSTI1/CASP1/ITGB7/GPR18/PSMB8/TAP1/PSMB9/PML/TNFAIP3/IRF7/PSME2/IFIH1/GCH1/PARP12/IDO1/FCGR1A/PARP14/PTPN6/CD74/IFI35/TRIM14/MX1/RIGI/USP18/CIITA/PSMB10/DDX60/IFI44/XAF1/CASP8/RTP4/CD274/FPR1/PTPN1/MVP/ZNFX1/CMPK2/IRF5/TAPBP/MARCHF1/TRIM25/CD86/RSAD2/DHX58/IL10RA/BATF2/TNFSF10/NFKBIA/WARS1/OAS2/CDKN1A/CXCL10/MX2/CCL2/UBE2L6/HLA-DMA/ISG20/LGALS3BP/IFI44L/RNF213/IFIT2/CSF2RB/OAS3/ISG15/OASL/ICAM1/HERC6/IFIT3/CCL7 |
| Up | Interferon alpha response | M5911 | 42 | 0.09524 | 1.22E-16 | SAMD9L/EPSTI1/CASP1/PSMB8/TAP1/PSMB9/IRF7/PARP9/PSME2/IFIH1/TMEM140/PARP12/PARP14/CD74/IFI35/TRIM14/MX1/USP18/DDX60/IFI44/UBA7/CASP8/RTP4/CMPK2/TRIM25/RSAD2/DHX58/BATF2/WARS1/CXCL10/SELL/GMPR/UBE2L6/CCRL2/ISG20/LGALS3BP/IFI44L/IFIT2/ISG15/OASL/HERC6/IFIT3 |
| Up | Inflammatory response | M5932 | 51 | 0.11565 | 1.34E-09 | LCP2/SLC1A2/TLR1/IRF7/IRAK2/GCH1/CD70/CD14/BTG2/NLRP3/IL18RAP/SPHK1/C3AR1/RTP4/FPR1/IL1A/P2RX7/CLEC5A/CSF3/OSM/TAPBP/CSF3R/CXCL8/NOD2/RGS1/SEMA4D/IL10RA/TNFRSF1B/MSR1/TNFSF10/NFKBIA/MXD1/HBEGF/CDKN1A/EREG/CXCL10/IL18R1/SELL/PTAFR/ACVR1B/CCL2/CCRL2/C5AR1/PIK3R5/IL1B/CCR7/PVR/SLC31A2/ICAM1/CYBB/CCL7 |
| Up | TNFa signaling via NFkB | M5890 | 49 | 0.11111 | 1.09E-08 | DUSP5/SLC16A6/TAP1/TNFAIP3/F2RL1/IFIH1/JAG1/GCH1/PLAU/DUSP4/LAMB3/BTG2/RIGI/YRDC/TRAF1/ETS2/B4GALT5/PLEK/MAFF/SPHK1/TNF/PLK2/IL1A/GADD45A/SDC4/CD80/TUBB2A/BIRC3/ZC3H12A/FOSL1/BMP2/FUT4/SERPINB2/BHLHE40/NFKBIA/MXD1/HBEGF/CDKN1A/CXCL10/AREG/NFKBIE/CCL2/CCRL2/PHLDA1/IL1B/TRIB1/IFIT2/IER2/ICAM1 |
| Up | Allograft rejection | M5950 | 47 | 0.10658 | 8.41E-08 | LCP2/IRF8/TLR1/TAP1/IRF7/TAP2/HLA-DOA/PTPN6/CCR1/CCR2/CD74/IGSF6/NLRP3/IL18RAP/PSMB10/LY86/TNF/LTB/GPR65/FGR/HLA-DRA/CD80/TAPBP/HLA-E/CTSS/ITGB2/CD86/PTPRC/HCLS1/FYB1/ITGAL/CCND2/WARS1/CFP/MMP9/EREG/NCF4/CCL2/HLA-DMA/IL1B/SRGN/IL2RG/HLA-DMB/HLA-DOB/ICAM1/CCL7/FCGR2B |
| Up | KRas signaling up | M5953 | 46 | 0.10431 | 2.08E-07 | IRF8/ADAM8/ARG1/CXCR4/PSMB8/TNFAIP3/F2RL1/DUSP6/PLAU/HSD11B1/MALL/YRDC/TRAF1/KCNN4/C3AR1/ALDH1A3/PLAT/BIRC3/CTSS/ITGB2/SOX9/BMP2/CA2/PLEK2/JUP/TLR8/WNT7A/IL10RA/TNFRSF1B/IKZF1/PRDM1/CCND2/HBEGF/MMP9/EREG/CXCL10/LCP1/MAFB/MMP10/CSF2RA/IL1B/TRIB1/IL2RG/GPNMB/CD37/DOCK2 |
| Up | p53 pathway | M5939 | 41 | 0.09297 | 2.62E-05 | RRAD/EPHA2/TRAF4/CASP1/DDB2/TAP1/TOB1/PCNA/BTG2/ZMAT3/CLCA2/BAX/SPHK1/PLK2/IL1A/GADD45A/ST14/HMOX1/XPC/BMP2/CDKN2B/PLK3/RAP2B/H1-2/RHBDF2/ITGB4/PTPN14/CCND2/DEF6/MXD1/HBEGF/CDKN1A/SERPINB5/ACVR1B/BAIAP2/PERP/SDC1/TM4SF1/AEN/KRT17/TGFA |
| Up | Apoptosis | M5902 | 29 | 0.06576 | 5.73E-03 | CASP1/SLC20A1/TAP1/GCH1/BCL2L1/CD14/BTG2/TSPO/BAX/PLCB2/TNF/CASP8/IL1A/GADD45A/PLAT/BIRC3/HMOX1/BMP2/HSPB1/KRT18/CDKN1B/TNFSF10/ANXA1/CCND2/RNASEL/CDKN1A/EREG/ISG20/IL1B |
| Up | IL6 JAK STAT3 signaling | M5897 | 18 | 0.04082 | 9.12E-03 | CD36/CCR1/CD14/TNF/LTB/PTPN1/HMOX1/CSF3R/TNFRSF1B/CXCL10/IL18R1/ACVR1B/PIK3R5/CSF2RA/IL1B/IL2RG/CSF2RB/CCL7 |
| Up | IL2 STAT5 signaling | M5947 | 31 | 0.07029 | 3.12E-02 | ITGA6/IRF8/ICOS/BCL2L1/CDC6/RHOH/TRAF1/MAFF/LTB/CDCP1/ITGAE/GPR65/TNFSF11/CD86/BMP2/CA2/COCH/IL10RA/TNFRSF1B/TNFSF10/BHLHE40/CCND2/MXD1/CXCL10/IL18R1/SELL/PHLDA1/MUC1/FURIN/CST7/SYNGR2 |
| Up | Complement | M5921 | 31 | 0.07029 | 3.12E-02 | LCP2/CASP1/DUSP5/ITGAM/PSMB9/TNFAIP3/IRF7/MMP13/CD36/SH2B3/DUSP6/CASP10/LTA4H/PLEK/CTSH/MAFF/MMP12/S100A9/SRC/PLAT/CTSS/CA2/PIK3CG/SERPINB2/CTSC/CDA/CTSB/PIK3R5/APOBEC3G/KCNIP2/F5 |
| Down | Epithelial mesenchymal transition | M5930 | 39 | 0.16525 | 1.97E-11 | LOXL1/FERMT2/PLOD2/SERPINH1/ENO2/THBS1/FAP/ABI3BP/APLP1/MEST/THY1/PLOD1/CALD1/IGFBP2/NT5E/VIM/LAMA1/SPOCK1/FN1/TPM2/BMP1/COL8A2/FBLN1/TNFRSF11B/TNC/VCAM1/SPARC/PRRX1/SCG2/HTRA1/TPM1/COL5A3/MYLK/COL12A1/TGFBR3/CDH11/MATN2/MATN3/CXCL12 |
| Down | UV response dn | M5942 | 19 | 0.08051 | 4.87E-03 | MAGI2/DLC1/PTGFR/NFIB/INPP4B/ATRX/MAP1B/KCNMA1/PLCB4/MMP16/CACNA1A/FYN/TGFBR3/PTPRM/APBB2/CDC42BPA/SYNE1/LPAR1/ADD3 |
| Down | Hypoxia | M5891 | 21 | 0.08898 | 3.33E-02 | GPC3/AKAP12/FAM162A/ENO2/EFNA1/WSB1/KDELR3/ZNF292/STC1/STC2/CSRP2/P4HA2/P4HA1/AK4/INHA/KLHL24/ALDOC/TMEM45A/PGF/CCN5/PGM1 |

**Supplemental Table 13 Enrichment analysis using MSigDB Hallmark at 14d**

| Group | Gene_set_name | ID | Count | GeneRatio | qvalue | GeneSymbol |
| --- | --- | --- | --- | --- | --- | --- |
| Up | TNFa signaling via NFkB | M5890 | 28 | 0.09790 | 3.0E-03 | INHBA/LDLR/F2RL1/DUSP4/LAMB3/F3/ETS2/B4GALT5/SPHK1/TNF/PFKFB3/GADD45A/TUBB2A/FOSL2/ZC3H12A/FOSL1/BMP2/FUT4/BHLHE40/CDKN1A/NFKBIE/IL1B/SPSB1/PLAUR/IL6/SMAD3/ICAM1/PMEPA1 |
| Up | p53 pathway | M5939 | 27 | 0.09441 | 3.8E-03 | EPHA2/TRAF4/DDB2/ZMAT3/BAX/SPHK1/GADD45A/ST14/XPC/BMP2/CDKN2B/RHBDF2/ITGB4/DEF6/CDKN1A/DDIT4/ACVR1B/BAIAP2/RPL18/TAX1BP3/SDC1/VDR/H2AJ/CDKN2A/AEN/KRT17/TGFA |
| Up | Apical junction | M5915 | 24 | 0.08392 | 2.3E-02 | CDH6/NECTIN4/COL17A1/LAMB3/MYH9/ACTB/CLDN7/CDH1/SRC/LAMA3/CLDN4/NECTIN1/JUP/ITGB4/ACTN1/MPZL2/NECTIN2/ACTN4/BAIAP2/FSCN1/GRB7/CDH3/ICAM1/MAPK11 |
| Up | E2F targets | M5925 | 24 | 0.08392 | 2.3E-02 | MELK/MCM4/CDK1/MYBL2/RRM2/CENPM/TK1/DCTPP1/MCM5/ESPL1/LIG1/AURKB/UBE2S/MCM2/CDKN1A/CDCA8/TCF19/NAA38/AURKA/HMGA1/ASF1B/CDKN2A/BIRC5/H2AX |
| Down | Myogenesis | M5909 | 69 | 0.23000 | 2.7E-31 | DES/MYF6/TNNT3/SGCD/ENO3/PYGM/CD36/ATP2A1/APLNR/DTNA/ACTC1/CSRP3/HSPB8/AK1/CAV3/NCAM1/CHRNB1/COX6A2/MAPK12/ACTA1/TNNC1/MYOM1/CACNG1/LAMA2/AGL/CASQ1/TPM2/MYH8/TNNI1/CASQ2/SSPN/MYL4/SVIL/MYH7/CKMT2/PDE4DIP/MYOG/MYL3/MYOZ1/LDB3/HSPB2/COX7A1/MYOM2/MYH11/MYL1/SPEG/FABP3/BIN1/MYLK/TNNT2/GJA5/PFKM/ACTN2/PKIA/ITGA7/RYR1/ACTN3/MYL11/TCAP/PTP4A3/MYL2/MB/MYH1/SGCA/CKM/PVALB/PGAM2/MEF2C/MYH4 |
| Down | UV response dn | M5942 | 20 | 0.06667 | 3.1E-02 | MAGI2/CAP2/AMPH/PTGFR/RGS4/DDAH1/TFPI/PLPP3/PLCB4/MMP16/NR3C1/CACNA1A/CDON/DAB2/FYN/TGFBR3/PTPRM/SYNE1/LPAR1/ADD3 |
| Down | KRas signaling up | M5953 | 25 | 0.08333 | 3.1E-02 | GPRC5B/AKAP12/GUCY1A1/KLF4/SPARCL1/CPE/TFPI/ITGBL1/SPON1/ADGRL4/CA2/PRRX1/PEG3/IL33/HDAC9/ABCB1/PECAM1/SCN1B/TNNT2/GFPT2/ALDH1A2/EPB41L3/GYPC/TMEM100/CFB |

**Supplemental Table 14 Top 30 of significantly changed genes at 3h after MMC treatment in HConFs**

| 3h-up |  | 3h-down |  |
| --- | --- | --- | --- |
| Gene Symbol | Fold Change | Gene Symbol | Fold Change |
| LOC101928093 | 99.33 | NPAS3 | 0.0294 |
| FAM71E2 | 92.72 | ATXN1 | 0.0916 |
| C1orf116 | 89.06 | KCTD16 | 0.0974 |
| LAMB2P1 | 51.15 | MED12L | 0.1241 |
| FNDC7 | 34.07 | FRMD5 | 0.1287 |
| KIAA1683 | 22.04 | FOXP2 | 0.1352 |
| RFPL3S | 14.54 | PALM2-AKAP2 | 0.1405 |
| PVRL4 | 14.37 | MAML3 | 0.1411 |
| BTG2 | 13.15 | ZFPM2 | 0.1538 |
| GPD1 | 11.82 | GLIS3 | 0.1633 |
| ALDH8A1 | 11.50 | MAGI1 | 0.1800 |
| BAAT | 9.57 | RBMS3 | 0.1920 |
| CLDN6 | 9.50 | THRB | 0.1935 |
| GABRR2 | 9.24 | NFATC2 | 0.1944 |
| AOC3 | 9.24 | BMPR1B | 0.2009 |
| DCAF4L1 | 8.95 | PDE3A | 0.2057 |
| PRKCG | 8.75 | SYT1 | 0.2105 |
| OVGP1 | 8.20 | SH3RF3 | 0.2155 |
| ACER2 | 6.98 | EYA2 | 0.2186 |
| TP53INP1 | 6.89 | SIPA1L2 | 0.2225 |
| SDPR | 6.71 | TMTC2 | 0.2236 |
| FAS-AS1 | 6.50 | HDAC4 | 0.2303 |
| TIPARP-AS1 | 6.39 | GPR39 | 0.2337 |
| AKR7L | 5.68 | JAZF1 | 0.2349 |
| GDF15 | 5.10 | KIAA1211 | 0.2375 |
| MDM2 | 4.99 | ENOX1 | 0.2502 |
| THSD1 | 4.96 | NAV2 | 0.2522 |
| B3GNT4 | 4.93 | PRDM16 | 0.2577 |
| ATF3 | 4.64 | CDKAL1 | 0.2600 |
| LSMEM1 | 4.64 | SNX29 | 0.2607 |

**Supplemental Table 15 Top 30 of significantly changed genes at 3d after MMC treatment in HConFs**

| 3d-up |  | 3d-down |  |
| --- | --- | --- | --- |
| Gene Symbol | Fold Change | Gene Symbol | Fold Change |
| SLC52A1 | 1051.85 | CDC20 | 0.0058 |
| CLCA2 | 619.29 | DLGAP5 | 0.0061 |
| FMO2 | 616.08 | TROAP | 0.0062 |
| BLNK | 481.96 | NEK2 | 0.0066 |
| PDE4C | 259.70 | SKA1 | 0.0070 |
| FAM13C | 257.48 | DEPDC1 | 0.0072 |
| BST2 | 225.56 | KIF14 | 0.0072 |
| TREM2 | 219.14 | SPC25 | 0.0074 |
| ADH1B | 213.32 | SGOL1 | 0.0075 |
| AGT | 192.72 | NUF2 | 0.0075 |
| LOC101927501 | 185.67 | HJURP | 0.0077 |
| ITIH5 | 184.96 | BIRC5 | 0.0081 |
| CYP3A7 | 154.30 | KIF20A | 0.0083 |
| ST8SIA1 | 153.14 | KIF18B | 0.0085 |
| SLPI | 143.71 | CENPA | 0.0093 |
| VTN | 143.47 | AURKB | 0.0096 |
| CASKIN1 | 134.94 | MKI67 | 0.0097 |
| C3 | 133.58 | BUB1B | 0.0100 |
| FCGR1A | 130.85 | UBE2C | 0.0101 |
| SLC1A7 | 130.56 | DEPDC1B | 0.0103 |
| C1orf95 | 128.14 | PLK1 | 0.0109 |
| SMPDL3B | 126.46 | KIF4A | 0.0110 |
| GOLGA8K | 118.77 | PBK | 0.0111 |
| LGI4 | 115.36 | HSPE1-MOB4 | 0.0112 |
| SULT1C4 | 115.07 | BUB1 | 0.0115 |
| CES3 | 109.98 | MYBL2 | 0.0117 |
| ETV7 | 107.77 | ZNF492 | 0.0118 |
| KIAA1683 | 102.56 | KIF2C | 0.0118 |
| DNAJC27-AS1 | 101.63 | CDC25C | 0.0121 |
| SULF2 | 97.19 | HMMR | 0.0122 |

**Supplemental Table 16 Top 30 of significantly changed genes at 14d after MMC treatment in HConFs**

| 14d-up |  | 14d-down |  |
| --- | --- | --- | --- |
| Gene Symbol | Fold Change | Gene Symbol | Fold Change |
| ITIH5 | 2587.73 | MKI67 | 0.0012 |
| DIRAS2 | 1208.66 | DLGAP5 | 0.0014 |
| FMO2 | 1197.01 | TROAP | 0.0015 |
| CLCA2 | 955.03 | KIF4A | 0.0017 |
| EGFL6 | 700.39 | CDC20 | 0.0019 |
| SFRP4 | 560.83 | DEPDC1 | 0.0020 |
| ADH1B | 559.96 | MYBL2 | 0.0020 |
| SLC52A1 | 518.34 | NEK2 | 0.0023 |
| BLNK | 489.66 | SKA1 | 0.0024 |
| SLC1A7 | 477.03 | BIRC5 | 0.0027 |
| ST8SIA1 | 466.34 | SPC25 | 0.0027 |
| RPTN | 441.72 | UBE2C | 0.0033 |
| SLPI | 431.09 | PBK | 0.0033 |
| PODN | 367.69 | CDK1 | 0.0034 |
| PCDHB16 | 353.25 | KIF20A | 0.0035 |
| AGT | 295.98 | KIF2C | 0.0035 |
| C3 | 287.42 | KIF18B | 0.0036 |
| OLR1 | 282.99 | NCAPH | 0.0038 |
| SCRG1 | 277.87 | NUF2 | 0.0038 |
| CCL7 | 276.77 | KIF14 | 0.0039 |
| FAM47E-STBD1 | 275.84 | RRM2 | 0.0041 |
| ADGRD1 | 275.56 | HMMR | 0.0046 |
| FAM13C | 271.73 | IQGAP3 | 0.0047 |
| TMEM178A | 268.03 | NCAPG | 0.0048 |
| RANBP3L | 262.19 | AURKB | 0.0049 |
| C1orf95 | 253.53 | FAM64A | 0.0050 |
| FCGR1A | 253.13 | CKAP2L | 0.0051 |
| MFAP5 | 241.12 | MCM10 | 0.0056 |
| THEMIS | 235.09 | BUB1B | 0.0057 |
| NRXN3 | 233.54 | BUB1 | 0.0058 |

**Supplemental Table 17 Enrichment analysis of MMC treated HConFs using MSigDB Hallmark at 3h**

| Group | Gene_set_name | ID | Count | GeneRatio | qvalue | GeneSymbol |
| --- | --- | --- | --- | --- | --- | --- |
| Up | p53_pathway | M5939 | 21 | 0.61765 | 3.87E-19 | AEN/ATF3/BTG2/CDK5R1/CDKN1A/FAS/FDXR/HEXIM1/IER5/MDM2/PHLDA3/PIDD1/PLK3/POLH/PPM1D/RNF19B/RRAD/SAT1/SESN1/TNFSF9/TOB1 |
| Up | TNFa_signaling_via_NFkB | M5890 | 8 | 0.23529 | 1.51E-03 | ATF3/BTG2/CDKN1A/FOSB/IER5/RNF19B/SAT1/TNFSF9 |
| Up | Apoptosis | M5902 | 6 | 0.17647 | 1.21E-02 | ATF3/BTG2/CDKN1A/FAS/FDXR/SAT1 |
| Down | UV_response_dn | M5942 | 10 | 0.21277 | 6.19E-05 | AKT3/APBB2/ATXN1/GRK5/INPP4B/PRKCE/RXRA/SIPA1L1/VAV2/ZMIZ1 |
| Down | Mitotic_spindle | M5893 | 9 | 0.19149 | 3.27E-03 | ARHGAP10/AURKA/CENPE/DOCK4/MYO1E/PIF1/PLK1/RASAL2/TIAM1 |
| Down | G2M_checkpoint | M5901 | 8 | 0.17021 | 1.16E-02 | AURKA/CENPA/CENPE/EFNA5/FOXN3/PLK1/RASAL2/STAG1 |

**Supplemental Table 18 Enrichment analysis of MMC treated HConFs using MSigDB Hallmark at 3d-up**

| Group | Gene_set_name | ID | Count | GeneRatio | qvalue | GeneSymbol |
| --- | --- | --- | --- | --- | --- | --- |
| Up | Interferon_gamma_response | M5913 | 85 | 0.13199 | 4.12E-21 | APOL6/B2M/BATF2/BST2/C1R/C1S/CASP1/CCL2/CD74/CDKN1A/CFB/CFH/CMPK2/DDX60/DHX58/EIF2AK2/EIF4E3/EPSTI1/FAS/FCGR1A/GBP4/GCH1/HELZ2/HERC6/HLA-A/HLA-B/HLA-DMA/HLA-G/ICAM1/IDO1/IFI27/IFI30/IFI35/IFIH1/IFIT1/IFIT2/IFIT3/IFITM2/IFITM3/IL18BP/IRF1/IRF7/IRF9/ISG15/ISG20/LGALS3BP/LY6E/MX1/MX2/NLRC5/NMI/OAS2/OAS3/PARP12/PARP14/PML/PSMB8/PSMB9/PTPN6/RNF213/RSAD2/RTP4/SAMD9L/SAMHD1/SECTM1/SERPING1/SOD2/SP110/SSPN/STAT1/STAT2/STAT3/STAT4/TAP1/TAPBP/TDRD7/TNFSF10/TRAFD1/TXNIP/UBE2L6/USP18/VAMP5/VAMP8/XAF1/ZNFX1 |
| Up | Interferon_alpha_response | M5911 | 54 | 0.08385 | 3.73E-20 | B2M/BATF2/BST2/C1S/CASP1/CD74/CMPK2/CSF1/DDX60/DHX58/EIF2AK2/EPSTI1/GBP2/GBP4/HELZ2/HERC6/HLA-C/IFI27/IFI30/IFI35/IFIH1/IFIT2/IFIT3/IFITM1/IFITM2/IFITM3/IRF1/IRF7/IRF9/ISG15/ISG20/LGALS3BP/LY6E/MOV10/MX1/NMI/OAS1/PARP12/PARP14/PARP9/PSMB8/PSMB9/RSAD2/RTP4/SAMD9L/SP110/STAT2/TAP1/TDRD7/TRAFD1/TXNIP/UBA7/UBE2L6/USP18 |
| Up | p53_pathway | M5939 | 71 | 0.11025 | 5.55E-13 | ABAT/ABHD4/ADA/AK1/ANKRA2/BLCAP/BTG2/CASP1/CCND2/CD82/CDKN1A/CEBPA/CLCA2/CTSD/CTSF/CYFIP2/DDB2/DEF6/DGKA/DRAM1/EPHX1/EPS8L2/FAS/FDXR/FOS/FUCA1/GLS2/GM2A/HSPA4L/IFI30/ISCU/ITGB4/JAG2/KRT17/MDM2/MKNK2/MXD4/NHLH2/NINJ1/NOTCH1/NUPR1/PHLDA3/PIDD1/PLK2/PLXNB2/POLH/PPM1D/RALGDS/RETSAT/RGS16/RHBDF2/RPS27L/RRAD/S100A4/SDC1/SESN1/SFN/STOM/TAP1/TM7SF3/TNFSF9/TOB1/TPD52L1/TRAF4/TRAFD1/TRIAP1/TSPYL2/TXNIP/VAMP8/VWA5A/ZMAT3 |
| Up | Epithelial_mesenchymal_transition | M5930 | 70 | 0.10870 | 1.44E-12 | ACTA2/APLP1/BGN/BMP1/CALD1/CAP2/CDH11/COL12A1/COL1A1/COL1A2/COL3A1/COL5A1/COL5A2/COL7A1/COL8A2/CTHRC1/CXCL12/DAB2/DCN/ECM1/EDIL3/EFEMP2/FAP/FAS/FBLN1/FBLN2/FBN1/FBN2/FMOD/FUCA1/GAS1/GJA1/GPC1/GPX7/HTRA1/IGFBP3/IGFBP4/IL32/LAMA3/LOX/LOXL1/LRP1/LUM/MAGEE1/MATN2/MEST/MFAP5/MGP/MMP2/MXRA5/MYL9/MYLK/NID2/NNMT/P3H1/PCOLCE/PDGFRB/SDC1/SFRP4/SLIT3/SPARC/TAGLN/TGFBI/TGFBR3/THBS1/THBS2/THY1/TIMP3/TNFRSF11B/VCAN |
| Up | Myogenesis | M5909 | 59 | 0.09161 | 2.07E-07 | ABLIM1/ACHE/ADCY9/AGRN/AK1/APOD/ATP2A1/ATP6AP1/BDKRB2/CD36/CDKN1A/CFD/CHRNB1/CKB/CLU/COL1A1/COL3A1/COX7A1/CRAT/CRYAB/CTF1/DMPK/EFS/ENO3/EPHB3/ERBB3/FABP3/FOXO4/FXYD1/GAA/GPX3/GSN/IGFBP3/ITGB4/LDB3/MAPRE3/MYLK/MYOM1/NOTCH1/PDE4DIP/PGAM2/PICK1/PLXNB2/PPFIA4/PPP1R3C/PTGIS/PTP4A3/PYGM/SGCA/SH3BGR/SOD3/SPARC/SPEG/SSPN/SYNGR2/TAGLN/TNNC1/TNNT3/TPD52L1 |
| Up | Apoptosis | M5902 | 44 | 0.06832 | 9.79E-05 | AIFM3/BCL2L10/BCL2L11/BGN/BMF/BNIP3L/BTG2/CASP1/CASP9/CCND2/CD14/CDKN1A/CFLAR/CLU/DCN/ERBB3/FAS/FDXR/FEZ1/GCH1/GPX1/GPX3/GPX4/GSN/IFITM3/IGFBP6/IRF1/ISG20/LGALS3/LUM/MMP2/PDCD4/PDGFRB/PPP2R5B/RETSAT/RNASEL/SATB1/SOD2/TAP1/TGFBR3/TIMP2/TIMP3/TNFSF10/TXNIP |
| Up | Coagulation | M5946 | 38 | 0.05901 | 2.67E-04 | A2M/ACOX2/ANG/BMP1/C1R/C1S/C2/C3/CASP9/CFB/CFD/CFH/CFI/CLU/CPQ/CTSH/CTSK/CTSO/F10/F8/FBN1/GP1BA/GSN/HTRA1/ISCU/LRP1/MASP2/MMP11/MMP2/MST1/OLR1/PROS1/RAPGEF3/RGN/SERPING1/SPARC/THBS1/TIMP3 |
| Up | Estrogen_response_early | M5906 | 47 | 0.07298 | 2.13E-03 | ABAT/ABCA3/ABLIM1/ADCY9/ADD3/ALDH3B1/ANXA9/AQP3/BLVRB/CELSR2/CLIC3/CXCL12/CYP26B1/DEPTOR/DHRS2/DHRS3/ELOVL2/FOS/FOXC1/FRK/GFRA1/GJA1/GREB1/IGFBP4/INPP5F/KAZN/KCNK15/KDM4B/KRT8/MUC1/NADSYN1/OLFML3/PEX11A/RAPGEFL1/RASGRP1/RET/RHOD/SEMA3B/SFN/SLC22A5/SLC37A1/SULT2B1/SYNGR1/TOB1/TPD52L1/TTC39A/WFS1 |
| Up | Bile_acid_metabolism | M5948 | 29 | 0.04503 | 4.73E-03 | ABCA1/ABCA2/ABCA3/ABCA4/ABCA8/ABCA9/ABCD1/ACSL5/AQP9/CROT/CYP27A1/CYP39A1/CYP46A1/EPHX2/FADS1/FDXR/HSD3B7/MLYCD/OPTN/PAOX/PEX11A/PEX11G/PEX6/PHYH/PRDX5/RBP1/RETSAT/SLC22A18/SULT2B1 |
| Up | Estrogen_response_late | M5907 | 44 | 0.06832 | 1.05E-02 | ABCA3/ACOX2/ADD3/ALDH3B1/ANXA9/ASS1/BLVRB/CACNA2D2/CELSR2/CKB/CLIC3/CPE/CXCL12/CXCL14/CYP26B1/DHRS2/FOS/FOXC1/FRK/GPER1/HSPA4L/IGFBP4/ISG20/MDK/MEST/PDCD4/PLXNB1/PTPN6/RAPGEFL1/RET/SCUBE2/SEMA3B/SERPINA3/SFN/SLC22A5/ST6GALNAC2/SULT2B1/TNNC1/TOB1/TPD52L1/TRIM29/TST/WFS1/ZFP36 |
| Up | Hypoxia | M5891 | 43 | 0.06677 | 1.66E-02 | ACKR3/BGN/BNIP3L/CDKN1A/CDKN1C/COL5A1/CP/DCN/DPYSL4/EFNA1/EFNA3/ENO3/FOS/GAA/GPC1/HEXA/IGFBP3/INHA/ISG20/KDELR3/KLHL24/LOX/MXI1/P4HA1/P4HA2/PGAM2/PGM1/PNRC1/PPARGC1A/PPFIA4/PPP1R3C/PRDX5/PYGM/S100A4/SDC2/SDC3/SELENBP1/SLC2A5/SULT2B1/TGFB3/TGFBI/TMEM45A/ZFP36 |
| Up | Angiogenesis | M5944 | 11 | 0.01708 | 3.43E-02 | CCND2/COL3A1/COL5A2/JAG2/KCNJ8/LRPAP1/LUM/OLR1/S100A4/VCAN/VTN |

**Supplemental Table 19 Enrichment analysis of MMC treated HConFs using MSigDB Hallmark at 3d-down**

| Group | Gene_set_name | ID | Count | GeneRatio | qvalue | GeneSymbol |
| --- | --- | --- | --- | --- | --- | --- |
| Down | E2F_targets | M5925 | 140 | 0.23689 | 8.00E-79 | ANP32E/ASF1B/ATAD2/AURKA/AURKB/BARD1/BIRC5/BRCA1/BRCA2/BUB1B/CCNB2/CDC20/CDC25A/CDCA3/CDCA8/CDK1/CDKN2C/CDKN3/CENPE/CENPM/CHEK1/CIT/CKS1B/CKS2/CSE1L/CTCF/CTPS1/DCK/DCLRE1B/DCTPP1/DDX39A/DEK/DEPDC1/DIAPH3/DLGAP5/DNMT1/DSCC1/DUT/E2F8/ESPL1/EXOSC8/EZH2/GINS1/GINS3/GINS4/HELLS/HMGA1/HMGB2/HMGB3/HMMR/HNRNPD/ILF3/ING3/IPO7/KIF18B/KIF22/KIF2C/KIF4A/KPNA2/LBR/LMNB1/LYAR/MAD2L1/MCM2/MCM3/MCM4/MCM5/MCM6/MCM7/MELK/MKI67/MMS22L/MSH2/MTHFD2/MXD3/MYBL2/MYC/NASP/NCAPD2/NOP56/NUP107/NUP153/NUP205/ORC6/PA2G4/PAICS/PHF5A/PLK1/PLK4/POLA2/POLD1/POLD2/POLD3/POLE/POP7/PRIM2/PSMC3IP/PTTG1/RACGAP1/RAD21/RAD51AP1/RAN/RANBP1/RBBP7/RFC2/RFC3/RNASEH2A/RPA3/RRM2/SHMT1/SLBP/SMC1A/SMC3/SMC4/SMC6/SNRPB/SPAG5/SPC24/SPC25/SRSF1/SRSF2/SSRP1/STMN1/SUV39H1/SYNCRIP/TACC3/TBRG4/TCF19/TIMELESS/TIPIN/TK1/TMPO/TOP2A/TRA2B/TRIP13/TUBB/UBE2S/UBE2T/UBR7/USP1 |
| Down | G2M_checkpoint | M5901 | 114 | 0.19289 | 2.37E-49 | AURKA/AURKB/BARD1/BIRC5/BRCA2/BUB1/BUB3/CCNA2/CCNB2/CCNF/CDC20/CDC25A/CDC45/CDC6/CDC7/CDK1/CDKN2C/CDKN3/CENPA/CENPE/CENPF/CHAF1A/CHEK1/CKS1B/CKS2/CTCF/DBF4/DDX39A/DKC1/DTYMK/E2F1/E2F2/ESPL1/EWSR1/EXO1/EZH2/FBXO5/GINS2/HMGA1/HMGB3/HMGN2/HMMR/HNRNPD/ILF3/INCENP/KIF11/KIF15/KIF20B/KIF22/KIF23/KIF2C/KIF4A/KPNA2/LBR/LMNB1/MAD2L1/MCM2/MCM3/MCM5/MCM6/MKI67/MT2A/MYBL2/MYC/NASP/NCL/NDC80/NEK2/NUSAP1/ODC1/ORC6/PBK/PLK1/PLK4/POLA2/POLE/POLQ/PRC1/PRIM2/PTTG1/PTTG3P/RACGAP1/RAD21/RAD54L/RBL1/RBM14/SAP30/SFPQ/SLC38A1/SLC7A1/SLC7A5/SMC1A/SMC2/SMC4/SNRPD1/SRSF1/SRSF2/STIL/STMN1/SUV39H1/SYNCRIP/TACC3/TFDP1/TLE3/TMPO/TOP2A/TPX2/TRA2B/TRAIP/TROAP/TTK/UBE2C/UBE2S/UCK2 |
| Down | Myc_targets_v1 | M5926 | 81 | 0.13706 | 2.26E-21 | ABCE1/BUB3/C1QBP/CBX3/CCNA2/CCT2/CCT4/CCT5/CDC20/CDC45/CDK2/CSTF2/CTPS1/DDX18/DDX21/DEK/DHX15/DUT/EIF1AX/EIF4A1/EIF4E/FBL/HNRNPA1/HNRNPA2B1/HNRNPA3/HNRNPC/HNRNPD/HNRNPR/HPRT1/HSP90AB1/HSPD1/HSPE1/KPNA2/LDHA/LSM2/MAD2L1/MCM2/MCM4/MCM5/MCM6/MCM7/MYC/NHP2/NOP16/NOP56/NPM1/ODC1/PA2G4/PHB2/POLD2/POLE3/PPM1G/PRPS2/PSMD14/PTGES3/RAN/RANBP1/RFC4/RNPS1/RRM1/RRP9/RUVBL2/SERBP1/SET/SNRPA/SNRPA1/SNRPD1/SNRPG/SRSF1/SRSF2/SRSF3/SRSF7/SSBP1/SYNCRIP/TCP1/TFDP1/TRA2B/TRIM28/U2AF1/UBA2/USP1 |
| Down | Myc_targets_v2 | M5928 | 37 | 0.06261 | 5.08E-18 | BYSL/CBX3/DCTPP1/DDX18/EXOSC5/GRWD1/HSPD1/HSPE1/IMP4/IPO4/MCM4/MCM5/MRTO4/MYC/NDUFAF4/NIP7/NOC4L/NOP16/NOP56/NPM1/PA2G4/PES1/PLK1/PLK4/PPAN/PRMT3/PUS1/RCL1/RRP12/RRP9/SLC19A1/SORD/TBRG4/TCOF1/TFB2M/UTP20/WDR74 |
| Down | Mitotic_spindle | M5893 | 55 | 0.09306 | 4.16E-07 | ANLN/ARHGDIA/AURKA/BIRC5/BRCA2/BUB1/CCNB2/CDC42EP1/CDK1/CDK5RAP2/CENPE/CENPF/CENPJ/CEP72/CNTRL/DLGAP5/DOCK2/ECT2/ESPL1/EZR/FBXO5/FGD4/INCENP/KIF11/KIF15/KIF20B/KIF22/KIF23/KIF2C/KIF4A/KNTC1/LMNB1/NDC80/NEDD9/NEK2/NUSAP1/PDLIM5/PIF1/PLK1/PRC1/PREX1/PXN/RACGAP1/RAPGEF5/SASS6/SMC1A/SMC3/SMC4/STK38L/TBCD/TOP2A/TPX2/TRIO/TTK/TUBGCP3 |
| Down | mTORC1_signaling | M5924 | 54 | 0.09137 | 1.09E-06 | ASNS/AURKA/BUB1/CACYBP/CCNF/CCT6A/CDC25A/CORO1A/CTH/DDIT4/DDX39A/DHFR/EBP/EEF1E1/ENO1/GMPS/GOT1/HMBS/HPRT1/HSPA9/HSPD1/HSPE1/LDHA/MAP2K3/MCM2/MCM4/MTHFD2/MTHFD2L/NUP205/PHGDH/PLK1/PNO1/PNP/POLR3G/PPP1R15A/PSAT1/PSMD14/PSMG1/PSPH/RRM2/RRP9/SHMT2/SLC1A5/SLC6A6/SLC7A11/SLC7A5/SORD/STC1/TOMM40/TPI1/TRIB3/TXNRD1/UCHL5/VLDLR |
| Down | Unfolded_protein_response | M5922 | 27 | 0.04569 | 1.03E-02 | ASNS/ATF4/BANF1/CEBPG/CHAC1/CKS1B/DDIT4/DDX10/DKC1/EIF4A1/EIF4E/EIF4EBP1/EXOSC2/EXOSC5/EXOSC9/FUS/HSPA9/KHSRP/LSM4/MTHFD2/NHP2/NOP56/NPM1/PSAT1/RRP9/SLC7A5/STC2 |
| Down | TNFa_signaling_via_NFkB | M5890 | 41 | 0.06937 | 1.67E-02 | AREG/ATP2B1/BCL2A1/BIRC2/DUSP5/EDN1/EIF1/F2RL1/FOSB/FOSL1/G0S2/GADD45B/GEM/GPR183/HBEGF/IER3/IL7R/INHBA/KLF10/MAFF/MAP2K3/MSC/MYC/NFKB1/PDLIM5/PHLDA1/PLAUR/PMEPA1/PPP1R15A/PTGS2/PTX3/RIPK2/SERPINB2/SERPINB8/SERPINE1/SGK1/SLC16A6/SPHK1/TGIF1/TIPARP/YRDC |

**Supplemental Table 20 Enrichment analysis of MMC treated HConFs using MSigDB Hallmark at 14d-up**

| Group | Gene_set_name | ID | Count | GeneRatio | qvalue | GeneSymbol |
| --- | --- | --- | --- | --- | --- | --- |
| Up | Epithelial_mesenchymal_transition | M5930 | 94 | 0.12737 | 7.37E-23 | ABI3BP/ACTA2/APLP1/BMP1/CADM1/CALD1/CALU/CAP2/CDH11/CDH6/COL11A1/COL12A1/COL1A1/COL1A2/COL3A1/COL4A1/COL5A1/COL5A2/COL8A2/CTHRC1/DAB2/DCN/ECM2/EDIL3/EFEMP2/FAP/FAS/FBLN1/FBLN2/FBN1/FBN2/FMOD/FN1/FSTL1/FUCA1/GAS1/GJA1/GPC1/GPX7/HTRA1/ID2/IGFBP2/IGFBP3/IGFBP4/IL15/IL32/LAMA1/LAMA2/LAMA3/LAMC1/LOX/LOXL1/LOXL2/LRP1/LUM/MAGEE1/MATN2/MEST/MFAP5/MGP/MMP2/MXRA5/MYL9/MYLK/NID2/NNMT/OXTR/PDGFRB/PLOD1/PLOD2/POSTN/QSOX1/SAT1/SCG2/SDC1/SFRP4/SGCD/SGCG/SLIT3/SPARC/SPOCK1/SPP1/TAGLN/TGFBI/TGFBR3/THBS1/THBS2/THY1/TIMP1/TIMP3/TNFRSF11B/TPM2/VCAM1/VCAN |
| Up | Myogenesis | M5909 | 70 | 0.09485 | 2.42E-09 | ABLIM1/ACHE/ADCY9/AGRN/AK1/APOD/APP/ATP6AP1/BDKRB2/CD36/CDKN1A/CFD/CKB/CLU/COL15A1/COL1A1/COL3A1/COX7A1/CRAT/CRYAB/CTF1/DMD/DMPK/DTNA/EPHB3/ERBB3/FABP3/FHL1/FOXO4/FXYD1/GAA/GPX3/GSN/HSPB2/IGFBP3/IGFBP7/ITGA7/LAMA2/LDB3/MAPRE3/MRAS/MYH11/MYH2/MYLK/MYOM1/MYOM2/NOS1/PDE4DIP/PGAM2/PICK1/PLXNB2/PPFIA4/PPP1R3C/PRNP/PSEN2/PTGIS/PTP4A3/RYR1/SGCD/SGCG/SH3BGR/SOD3/SPARC/SPEG/SSPN/SYNGR2/TAGLN/TNNC2/TPD52L1/TPM2 |
| Up | Interferon_gamma_response | M5913 | 68 | 0.09214 | 1.27E-08 | APOL6/B2M/BANK1/BST2/C1R/C1S/CCL2/CCL7/CD40/CD74/CDKN1A/CFB/CFH/CXCL10/DDX60/DHX58/EIF4E3/FAS/FCGR1A/GBP4/GCH1/HERC6/HLA-A/HLA-B/HLA-DMA/HLA-DRB1/HLA-G/ICAM1/IDO1/IFI27/IFI35/IFIH1/IFIT1/IFIT2/IFIT3/IFITM3/IL15/IL7/IRF1/IRF9/ISG15/LGALS3BP/MX1/MX2/OAS2/PARP14/PML/PSMB9/RTP4/SAMD9L/SERPING1/SOD2/SSPN/ST3GAL5/STAT1/STAT2/STAT3/STAT4/TAP1/TAPBP/TDRD7/TNFSF10/TOR1B/UBE2L6/VAMP8/VCAM1/XAF1/ZNFX1 |
| Up | Interferon_alpha_response | M5911 | 37 | 0.05014 | 2.77E-06 | B2M/BST2/C1S/CD74/CSF1/CXCL10/DDX60/DHX58/GBP2/GBP4/GMPR/HERC6/HLA-C/IFI27/IFI35/IFIH1/IFIT2/IFIT3/IFITM1/IFITM3/IL15/IL7/IRF1/IRF9/ISG15/LGALS3BP/MX1/PARP14/PARP9/PSMB9/RTP4/SAMD9L/STAT2/TAP1/TDRD7/UBA7/UBE2L6 |
| Up | p53_pathway | M5939 | 59 | 0.07995 | 2.58E-05 | ABAT/ABHD4/ADA/AK1/ANKRA2/APP/BLCAP/BTG2/CCND2/CD81/CD82/CDKN1A/CLCA2/CTSD/CTSF/CYFIP2/DGKA/DRAM1/EPHX1/EPS8L2/FAS/FDXR/FUCA1/GM2A/HSPA4L/INHBB/ISCU/JAG2/KRT17/MDM2/MXD4/NHLH2/NINJ1/NUPR1/PERP/PHLDA3/PIDD1/PLK2/PLXNB2/POLH/RALGDS/RETSAT/RHBDF2/RRAD/SAT1/SDC1/SESN1/STOM/TAP1/TCHH/TM7SF3/TNFSF9/TOB1/TP63/TPD52L1/TSPYL2/VAMP8/VWA5A/ZMAT3 |
| Up | Coagulation | M5946 | 44 | 0.05962 | 4.43E-05 | ACOX2/ANG/ARF4/BMP1/C1R/C1S/C2/C3/CASP9/CFB/CFD/CFH/CFI/CLU/CPQ/CTSB/CTSH/CTSK/CTSO/DPP4/F10/F8/FBN1/FN1/GP1BA/GSN/HTRA1/ISCU/LGMN/LRP1/MMP11/MMP15/MMP2/MST1/OLR1/PROS1/PRSS23/RAPGEF3/RGN/SERPING1/SPARC/THBS1/TIMP1/TIMP3 |
| Up | Angiogenesis | M5944 | 15 | 0.02033 | 1.80E-03 | APP/CCND2/COL3A1/COL5A2/FSTL1/JAG2/KCNJ8/LRPAP1/LUM/OLR1/POSTN/SPP1/TIMP1/VCAN/VTN |
| Up | Apoptosis | M5902 | 42 | 0.05691 | 7.17E-03 | APP/BCL2L11/BNIP3L/BTG2/CASP9/CCND1/CCND2/CD14/CDKN1A/CFLAR/CLU/DCN/ERBB3/FAS/FDXR/FEZ1/GCH1/GPX3/GSN/IFITM3/IFNGR1/IGF2R/IGFBP6/IL18/IRF1/LUM/MMP2/PDCD4/PDGFRB/PPP2R5B/PSEN2/RETSAT/RNASEL/SAT1/SATB1/SOD2/TAP1/TGFBR3/TIMP1/TIMP2/TIMP3/TNFSF10 |
| Up | Estrogen_response_early | M5906 | 49 | 0.06640 | 1.14E-02 | ABAT/ABCA3/ABLIM1/ADCY9/ADD3/ANXA9/CALB2/CCND1/CELSR1/CELSR2/CLIC3/CYP26B1/DEPTOR/DHRS2/DHRS3/ELF3/ELOVL2/FOXC1/FRK/GAB2/GFRA1/GJA1/GREB1/IGFBP4/INHBB/KAZN/KCNK15/KRT8/MUC1/NADSYN1/NBL1/OLFM1/OLFML3/PAPSS2/PRSS23/RASGRP1/RET/RHOD/SEMA3B/SH3BP5/SLC1A1/SLC22A5/SLC37A1/SYBU/SYNGR1/TOB1/TPD52L1/TTC39A/WFS1 |
| Up | Bile_acid_metabolism | M5948 | 30 | 0.04065 | 1.68E-02 | ABCA1/ABCA2/ABCA3/ABCA4/ABCA5/ABCA8/ABCA9/ABCD2/ACSL5/ALDH8A1/AMACR/AQP9/CROT/CYP27A1/CYP39A1/CYP46A1/CYP7B1/DIO2/EPHX2/FDXR/HSD17B6/MLYCD/OPTN/PEX11G/PIPOX/PRDX5/RBP1/RETSAT/SCP2/SLC22A18 |
| Up | Xenobiotic_metabolism | M5934 | 47 | 0.06369 | 2.61E-02 | ABCD2/ACOX1/ACOX2/AKR1C2/AKR1C3/ALDH2/APOE/AQP9/CD36/CDO1/CFB/CROT/CSAD/CYFIP2/CYP27A1/CYP2E1/DHRS1/ECH1/EPHX1/F10/FAS/FBLN1/FETUB/FMO1/FMO3/GABARAPL1/GCH1/ID2/IGFBP4/ITIH4/JUP/MAN1A1/MPP2/NDRG2/NINJ1/PAPSS2/PINK1/PROS1/PTGDS/RAP1GAP/RETSAT/SLC12A4/SLC46A3/SPINT2/TDO2/TTPA/VTN |

**Supplemental Table 21 Enrichment analysis of MMC treated HConFs using MSigDB Hallmark at 14d-down**

| Group | Gene_set_name | ID | Count | GeneRatio | qvalue | GeneSymbol |
| --- | --- | --- | --- | --- | --- | --- |
| Down | E2F_targets | M5925 | 149 | 0.22305 | 2.89E-82 | ANP32E/ASF1A/ASF1B/ATAD2/AURKA/AURKB/BARD1/BIRC5/BRCA1/BRCA2/BUB1B/CCNB2/CCP110/CDC20/CDC25A/CDCA3/CDCA8/CDK1/CDKN2C/CDKN3/CENPE/CENPM/CHEK1/CIT/CKS1B/CKS2/CSE1L/CTCF/CTPS1/DCK/DCLRE1B/DCTPP1/DDX39A/DEK/DEPDC1/DIAPH3/DLGAP5/DNMT1/DONSON/DSCC1/DUT/E2F8/ESPL1/EXOSC8/EZH2/GINS1/GINS3/GINS4/HELLS/HMGA1/HMGB2/HMGB3/HMMR/HNRNPD/ILF3/ING3/IPO7/KIF18B/KIF22/KIF2C/KIF4A/KPNA2/LBR/LIG1/LMNB1/LYAR/MAD2L1/MCM2/MCM3/MCM4/MCM5/MCM6/MCM7/MELK/MKI67/MMS22L/MSH2/MTHFD2/MXD3/MYBL2/MYC/NASP/NBN/NCAPD2/NME1/NOLC1/NOP56/NUP107/NUP153/NUP205/ORC6/PA2G4/PAICS/PDS5B/PHF5A/PLK1/PLK4/POLA2/POLD1/POLD2/POLD3/POLE/PRIM2/PRKDC/PSIP1/PSMC3IP/PTTG1/RACGAP1/RAD21/RAD51AP1/RAN/RANBP1/RBBP7/RFC2/RFC3/RNASEH2A/RPA3/RRM2/SHMT1/SMC1A/SMC3/SMC4/SMC6/SNRPB/SPAG5/SPC24/SPC25/SRSF1/SRSF2/SSRP1/STMN1/SUV39H1/SYNCRIP/TACC3/TBRG4/TCF19/TIMELESS/TIPIN/TK1/TMPO/TOP2A/TRA2B/TRIP13/TUBB/UBE2S/UBE2T/UBR7/USP1/WDR90 |
| Down | G2M_checkpoint | M5901 | 128 | 0.19162 | 1.53E-57 | AURKA/AURKB/BARD1/BIRC5/BRCA2/BUB1/BUB3/CASP8AP2/CCNA2/CCNB2/CCNF/CDC20/CDC25A/CDC45/CDC6/CDC7/CDK1/CDKN2C/CDKN3/CENPA/CENPE/CENPF/CHAF1A/CHEK1/CKS1B/CKS2/CTCF/DBF4/DDX39A/DKC1/DTYMK/E2F1/E2F2/E2F4/ESPL1/EWSR1/EXO1/EZH2/FANCC/FBXO5/GINS2/HMGA1/HMGB3/HMGN2/HMMR/HNRNPD/HNRNPU/ILF3/INCENP/KIF11/KIF15/KIF20B/KIF22/KIF23/KIF2C/KIF4A/KPNA2/LBR/LIG3/LMNB1/MAD2L1/MCM2/MCM3/MCM5/MCM6/MEIS1/MKI67/MT2A/MTF2/MYBL2/MYC/NASP/NCL/NDC80/NEK2/NOLC1/NUP50/NUSAP1/ODC1/ODF2/ORC6/PBK/PDS5B/PLK1/PLK4/POLA2/POLE/POLQ/PRC1/PRIM2/PTTG1/PTTG3P/RACGAP1/RAD21/RAD54L/RBL1/RBM14/SAP30/SFPQ/SLC38A1/SLC7A1/SLC7A5/SMARCC1/SMC1A/SMC2/SMC4/SNRPD1/SRSF1/SRSF2/STIL/STMN1/SUV39H1/SYNCRIP/TACC3/TFDP1/TGFB1/TLE3/TMPO/TOP1/TOP2A/TPX2/TRA2B/TRAIP/TROAP/TTK/UBE2C/UBE2S/UCK2 |
| Down | Myc_targets_v1 | M5926 | 96 | 0.14371 | 2.94E-28 | ABCE1/BUB3/C1QBP/CBX3/CCNA2/CCT2/CCT4/CCT5/CCT7/CDC20/CDC45/CDK2/CSTF2/CTPS1/CYC1/DDX18/DDX21/DEK/DHX15/DUT/EIF1AX/EIF4A1/FBL/HDAC2/HDGF/HNRNPA1/HNRNPA2B1/HNRNPA3/HNRNPC/HNRNPD/HNRNPR/HNRNPU/HPRT1/HSP90AB1/HSPD1/HSPE1/IMPDH2/KPNA2/LSM2/MAD2L1/MCM2/MCM4/MCM5/MCM6/MCM7/MRPL23/MYC/NHP2/NME1/NOLC1/NOP16/NOP56/NPM1/ODC1/PA2G4/PHB2/POLD2/POLE3/PPM1G/PRPS2/PTGES3/RAN/RANBP1/RFC4/RNPS1/RPL14/RPL18/RPS2/RPS3/RPS5/RRM1/RRP9/RUVBL2/SERBP1/SET/SF3B3/SMARCC1/SNRPA/SNRPA1/SNRPB2/SNRPD1/SNRPG/SRSF1/SRSF2/SRSF3/SRSF7/SSBP1/SYNCRIP/TCP1/TFDP1/TRA2B/TRIM28/TYMS/U2AF1/UBA2/USP1 |
| Down | Myc_targets_v2 | M5928 | 38 | 0.05689 | 3.33E-17 | BYSL/CBX3/DCTPP1/DDX18/DUSP2/EXOSC5/GRWD1/HSPD1/HSPE1/IMP4/IPO4/MCM4/MCM5/MRTO4/MYC/NDUFAF4/NIP7/NOC4L/NOLC1/NOP16/NOP2/NOP56/NPM1/PA2G4/PES1/PLK1/PLK4/PPAN/PRMT3/PUS1/RCL1/RRP9/SLC19A1/SORD/TBRG4/TCOF1/UTP20/WDR74 |
| Down | Mitotic_spindle | M5893 | 55 | 0.08234 | 2.59E-05 | ANLN/ARHGDIA/AURKA/BIRC5/BRCA2/BUB1/CCNB2/CDC42EP1/CDK1/CDK5RAP2/CENPE/CENPF/CENPJ/CEP72/CNTRL/CNTROB/DLGAP5/DOCK2/ECT2/ESPL1/EZR/FBXO5/GEMIN4/INCENP/KIF11/KIF15/KIF20B/KIF22/KIF23/KIF2C/KIF4A/KNTC1/LMNB1/MID1IP1/NDC80/NEK2/NET1/NUSAP1/PCNT/PIF1/PLK1/PRC1/PXN/RACGAP1/RAPGEF6/SASS6/SMC1A/SMC3/SMC4/STK38L/TOP2A/TPX2/TRIO/TTK/TUBGCP3 |
| Down | mTORC1_signaling | M5924 | 49 | 0.07335 | 2.15E-03 | ASNS/AURKA/BUB1/CACYBP/CCNF/CCT6A/CDC25A/CORO1A/CTH/DDIT4/DDX39A/DHFR/EBP/EEF1E1/ENO1/GMPS/GOT1/HMBS/HPRT1/HSPD1/HSPE1/MAP2K3/MCM2/MCM4/MTHFD2/MTHFD2L/NFIL3/NUP205/PDAP1/PHGDH/PITPNB/PLK1/PNP/POLR3G/PPP1R15A/PSMG1/PSPH/RRM2/RRP9/SHMT2/SLC1A5/SLC6A6/SLC7A11/SLC7A5/SORD/STC1/TOMM40/TRIB3/UCHL5 |
| Down | Unfolded_protein_response | M5922 | 29 | 0.04341 | 1.48E-02 | ASNS/ATF4/BANF1/CEBPG/CHAC1/CKS1B/CXXC1/DDIT4/DDX10/DKC1/EIF4A1/EIF4A3/EIF4EBP1/EXOSC10/EXOSC2/EXOSC5/EXOSC9/FUS/GEMIN4/KHSRP/MTHFD2/NFYA/NHP2/NOLC1/NOP56/NPM1/RRP9/SLC7A5/ZBTB17 |
| Down | TNFa_signaling_via_NFkB | M5890 | 45 | 0.06737 | 1.82E-02 | AREG/ATP2B1/BCL2A1/BIRC2/BTG1/CLCF1/DUSP2/DUSP5/ETS2/F2RL1/FOSB/FOSL1/G0S2/GADD45B/GEM/GPR183/HBEGF/IER2/IER3/IL7R/INHBA/KLF10/KLF4/KLF6/MAFF/MAP2K3/MCL1/MYC/NFIL3/NFKB1/NR4A1/NR4A2/PHLDA1/PLAU/PLAUR/PMEPA1/PPP1R15A/PTGS2/PTX3/RIPK2/SERPINB8/SPHK1/TGIF1/TIPARP/YRDC |
